# Towards Nematic Phases in Ionic Liquid Crystals – A Simulation Study

**DOI:** 10.1002/cphc.202200424

**Published:** 2022-10-25

**Authors:** Christian Haege, Stefan Jagiella, Frank Giesselmann

**Affiliations:** ^1^ Institute of Physical Chemistry University of Stuttgart Pfaffenwaldring 55 70569 Stuttgart Germany

**Keywords:** ionic liquid crystals, molecular dynamics, phase behaviour, charge position, nematic phases

## Abstract

Ionic liquid crystals (ILCs) are soft matter materials with broad liquid crystalline phases and intrinsic electric conductivity. They typically consist of a rod‐shaped mesogenic ion and a smaller spherical counter‐ion. Their mesomorphic properties can be easily tuned by exchanging the counter ion. ILCs show a strong tendency to form smectic A phases due to the segregation of ionic and the non‐ionic molecular segments. Nematic phases are therefore extremely rare in ILCs and the question of why nematic phases are so exceptional in existing ILCs, and how nematic ILCs might be obtained in the future is of vital interest for both the fundamental understanding and the potential applications of ILCs. Here, we present the result of a simulation study, which highlights the crucial role of the location of the ionic charge on the rod‐like mesogenic ions in the phase behaviour of ILCs. We find that shifting the charge from the ends towards the centre of the mesogenic ion destabilizes the liquid crystalline state and induces a change from smectic A to nematic phases.

## Graphical abstract

**The influence of charge location** in a mesogenic unit on the phase behaviour of an ionic liquid crystal is investigated using coarse grained molecular dynamics on a simple model system.The phase behaviour is found to be greatly influenced by the location of charge and a phase change occurs at a certain displacement of the charge from the centre of the mesogenic ion.

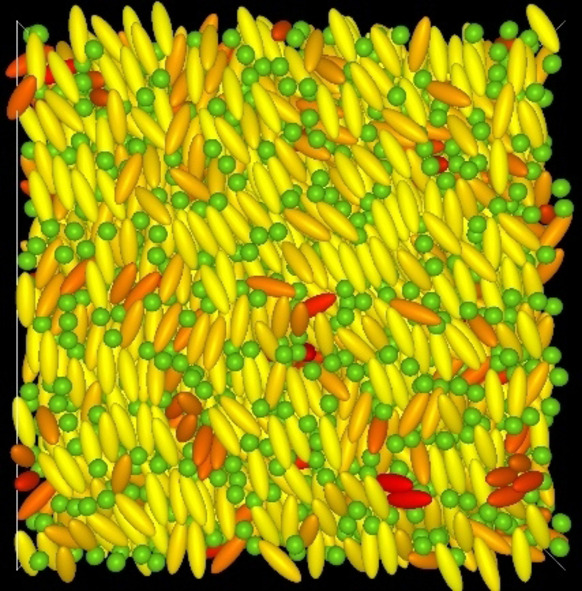

## Introduction

Ionic liquid crystals (ILCs) are liquid crystals typically consisting of large rod‐shaped organic ions and smaller and more spherical counter ions, the latter not necessarily being organic. They have broad liquid crystalline phases, intrinsic electric conductivity and their mesomorphic properties can be tuned by exchanging the counter ion.[^1^, ^2^] These properties combined make them interesting for many potential applications.^[3]^

**Figure 1:**
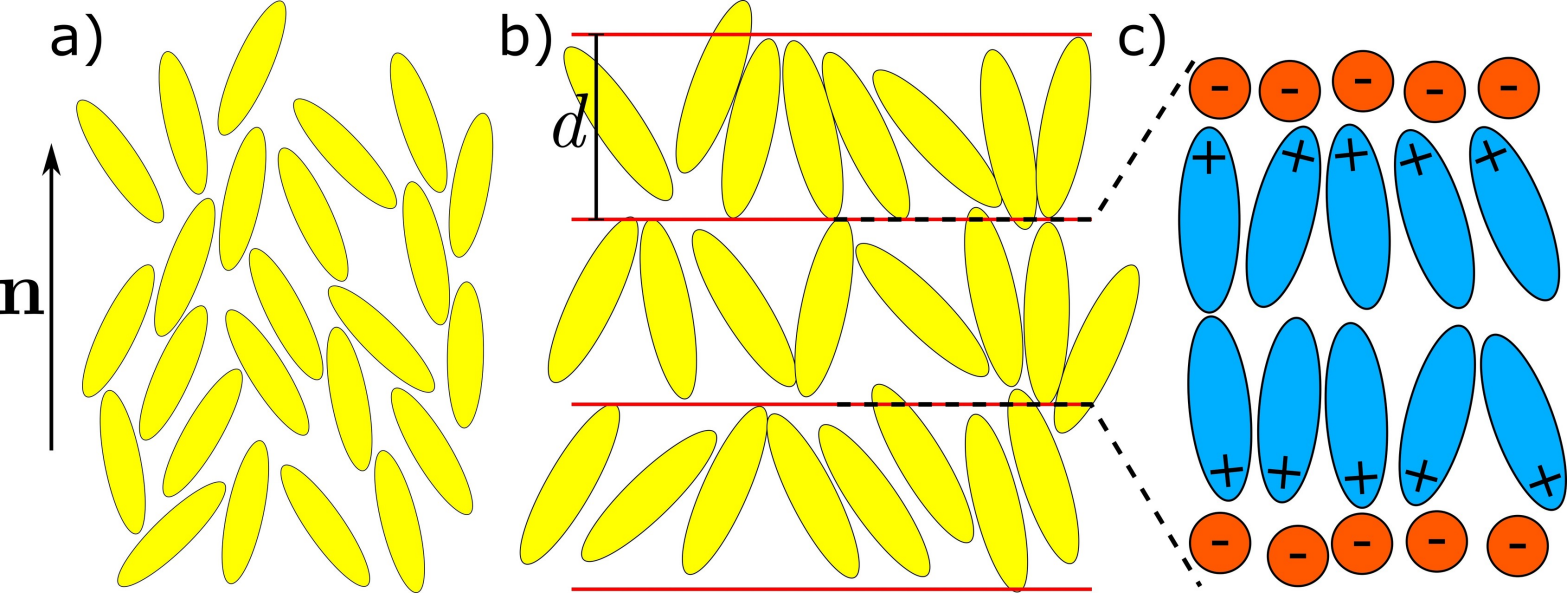
a) Schematic of a nematic phase with director n . The director also applies to subfigures b) and c). b) Schematic of a smectic A phase with layer thickness d . c) Zoom into a smectic layer of an ionic liquid crystal. The layer is segregated into ions and counter ions.

The simplest and archetypical example of a liquid crystal (LC) phase is the nematic (N) phase, which has orientational long‐range order (LRO) of the principal molecular axis (i. e., the long molecular axis in the case of rod‐like mesogenic molecules, see Figure 1a) along a certain direction, called the director n , but no kind of translational LRO. While nematic phases are frequently found in many non‐ionic thermotropic LCs, they are almost never found in ILCs. Instead, ILCs show a strong tendency to form layered smectic phases, normally smectic A (SmA) phases.^[2]^ In addition to orientational LRO of nematic phases, smectic phases possess 1D‐translational LRO with a certain period d . They can be understood as a 1D periodic stack of 2D‐fluid molecular layers (Figure 1b). The SmA phases of ILCs consist of bilayers with the ionic and the non‐ionic segments of the ILC building blocks segregated into different sub‐layers^[2]^ (Figure 1c). This segregation is assumed to be the driving force for the formation of smectic phases in ILCs,^[2]^ likewise to the segregation into hydrophilic and hydrophobic sublayers found in the case of lyotropic lamellar phases.^[4]^

**Figure 2:**
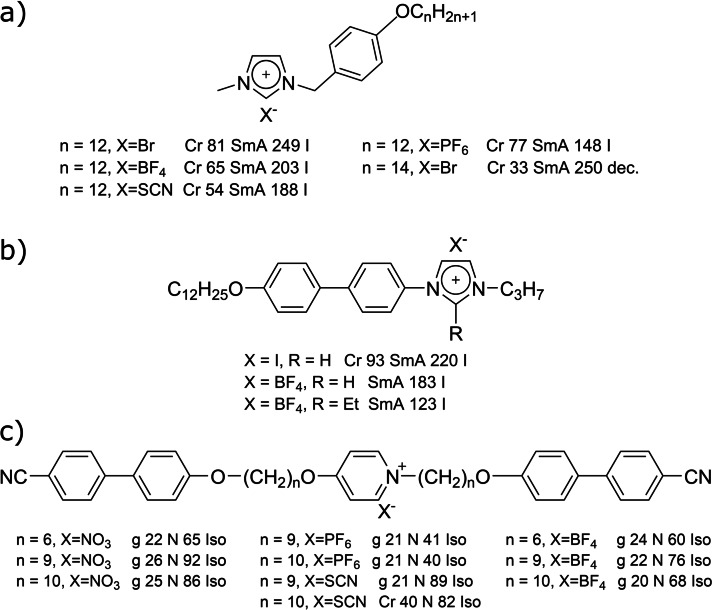
a‐b) Examples of typical imidazolium based ionic liquid crystals with transition temperatures.^[5]^ c) A few of the rare examples of ionic liquid crystals with nematic phases.[^6^, ^7^]

Selected examples of ILC molecular structures are shown in Figure 2. In typical examples such as the widely investigated imidazolium based ILCs in Figure 2a–b the positive charge is located at or close to the termini of the mesogenic unit. All these materials have broad SmA phases but no nematic phases. Nevertheless, very few exceptions[^6^, ^7^, ^8^] of ILCs with nematic phases have been reported, an example of which is shown in Figure 2c. On the one hand these examples have cyanobyphenyl groups at their tips, which is also part of the famous nematic 5CB (4‐Cyano‐4′‐pentylbiphenyl),^[9]^ on the other hand the charge is more or less located at the centre of the mesogenic ion, which hinders the segregation into ionic and non‐ionic sublayers as shown in Figure 1c, destabilizing the smectic phase. It therefore raises the question: Is the location of charge one of the keys to new nematic ILCs?

The question of why nematic phases are so exceptional in existing ILCs and how nematic ILCs might be obtained in the future is of vital interest for both the fundamental understanding and the potential applications of ILCs, since nematic phases are less viscous and easier to align then the smectic phases common in present ILCs.

We here present the results of a simulation study, which highlights the crucial role of the location of the charge on the formation of nematic phases in ILCs. To test our hypothesis, whether the location of charge is one of the keys for nematic behaviour in ILCs, we start from a simulation by Saielli et al.,[^10^, ^11^] which is known to show nematic phases and has the charge located right at the centre of the mesogenic ion. The novelty of our study is that we move the charge incrementally from the centre closer to the tip of the mesogenic ion, a scenario which is hardly possible in a real system. With this model system we separate the influence of the location of charge from other parameters. If our hypothesis is correct, moving the charge away from the centre should lead to a change from nematic to smectic mesomorphism.

## Model

**Figure 3:**
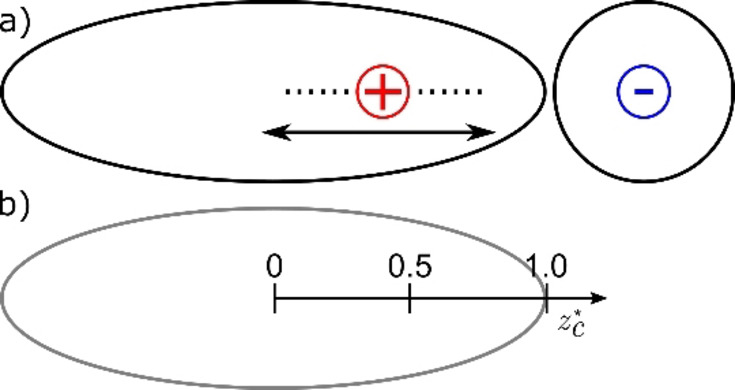
a) Schematic of a charged Gay‐Berne (GB) particle and an oppositely charged Lennard‐Jones (LJ) particle. The charge position is varied along the long axis of the GB particles between different simulation runs. b) Definition of the reduced charge position on a GB particle.

We simulate 1 : 1 mixtures of oppositely charged anisotropic Gay‐Berne^[12]^ (GB) particles with spherical Lennard‐Jones (LJ) particles (see Figure 3a), where the position/location of the charge on the GB particle is varied between different simulation runs. The reduced charge position $z_{c}^{{}^{*}}$ is given by

(1) $$z_{c}^{{}^{*}}=\frac{z_{c}}{r_{z,GB}}$$

with $r_{z,GB}$ the long radius of a GB particle and $z_{c}$ the charge position along the long axis of a GB particle. As shown in Figure 3b, the centre of the GB particle corresponds to a relative charge position of $z_{c}^{{}^{*}}=0$ .

## Simulation Parameters

Using the ESPResSo^[13]^ package for molecular dynamic simulations we simulate a total of 5416 GB and 5416 LJ particles using an NVT ensemble, a Langevin thermostat,[^13^, ^14^] a reduced timestep of $\Delta t^{{}^{*}}=0.0015$ and a number density of $\rho^{{}^{*}}=0.44716$ . The LJ and GB particles carry a reduced charge of $q^{{}^{*}}=\pm 2$ of opposite sign. The number density, the reduced charge and the number of particles are the same as in some simulations from Saielli et al.[^10^, ^11^] For the typical dimensions of a LC molecule like 4,4’‐dimethoxyazoxybenzene the reduced charge of $q^{{}^{*}}=\pm 2$ corresponds to an effective charge of q=±e/5 , where e is the elementary charge. This is calculated using $q^{{}^{*}}=\;{\left(4\pi \epsilon_{0}\epsilon_{0}\sigma_{0}\right)}^{-1/2}q$ from Ref. [11], where $\epsilon_{0}$ is the vacuum permittivity and $\epsilon_{0}=\;3.2\;\;\mathrm{kJ}/\mathrm{mol}$ and $\sigma_{0}=4.5\;\;Å$ are scaling factors from Ref. [15] for 4,4’‐dimethoxyazoxybenzene. This has been discussed in detail by Saielli et al.;^[11]^ the ratio of the potentials non‐ionic (GB) to ionic interaction strength is in a realistic range, which leads to the formation of liquid crystalline phases.

The parameters of the GB interaction are the same as the ones used by Berardi et al.^[16]^ and Saielli et al.[^10^, ^11^] The GB‐to‐GB interaction is modelled using the ratio of the diameters of the semi‐major axes $k_{1}=3$ , the ratio of the potentials side‐by‐side and end‐to‐end configurations well depths $k_{2}=5$ , the adjustable exponents μ=1 and ν=3 , the diameter of the semi‐minor axis $\sigma_{0}=1$ , the well depth of the end‐to‐end configuration $\epsilon_{0}=1$ . The exact equations of the GB‐potential can be found in the SI. The LJ‐to‐LJ interaction uses parameters σ=1 and ϵ=1 . Here σ is the diameter of the LJ particle and ϵ is the potentials well depth. The LJ potential is the classic 12‐6 potential. For reasons of simplicity, the parameters of the interaction between the GB and the LJ particles are calculated by Lorentz^[17]^ and Berthelot^[18]^ mixing rules.^[19]^ It is thus modelled like a GB interaction with parameters $k_{1}=2$ , $k_{2}=\sqrt{5}$ , μ=1 , ν=3 , $\sigma_{0}=1$ , $\epsilon_{0}=1$ . Effectively the mixed interaction of the spherical and ellipsoidal particle is modelled by two ellipsoidal particles of intermediate size and potential. The slight overlap of ellipsoidal and spherical particles sometimes seen in the snapshots (see Figure 5a or SI) is an artifact of the application of the mixing rules. For a detailed explanation, see Figure S1 of the SI.

While the LJ particle is charged, the charge of the GB particle is added via a virtual particle with charge $q^{{}^{*}}$ at $z_{c}^{{}^{*}}$ . All forces that act on the virtual particle are projected on the GB particle. In a series of simulation runs, the position of the virtual particle, and thus the location of the charge on the GB particle, is systematically varied along the long axis of the GB particle (see also Figure 3). Further details about the simulation procedure are found in the SI.

## Simulation Analysis

The orientational order parameter $S_{2}$ , given by

(2) $$S_{2}=\frac{1}{2}\left(3{\left\langle \cos^{2}\beta_{i}\right\rangle }_{i}-1\right),$$

is a scalar, which measures the quality of long‐range orientational order. $\beta_{i}$ is the angle between the long axis of a mesogen i and the director n . For an isotropic phase $S_{2}=0$ and for a liquid crystalline phase $0<S_{2}<1$ . In this paper values of the orientational order parameter are determined by diagonalizing the ordering tensor Q . The largest eigenvalue of Q is $S_{2}$ and the eigenvector of that eigenvalue is n .[^20^, ^21^] The degree of translational long‐range order can be quantified by the 1D‐translational order parameter Σ , which is given by

(3) $$\Sigma =\;{\left\langle \cos\frac{2\pi z_{i}}{d}\right\rangle }_{i}.$$

Σ=0 applies to isotropic and nematic phases, 0<Σ<1 to SmA phases.^[22]^ Here d is the smectic layer repeat period and $z_{i}$ is the projection of a mesogens coordinate on to n . Σ and d are calculated by iteratively maximizing Σ in Equation 4:^[20]^

(4) $$\Sigma =\sqrt{{\left\langle \cos\frac{2\pi z_{i}}{d}\right\rangle }_{i}^{2}+{\left\langle \sin\frac{2\pi z_{i}}{d}\right\rangle }_{i}^{2}}.$$

Whether the resulting phases are polar is checked using the first Legendre polynomial of $\left\langle \cos\beta_{i}\right\rangle$ , which is given by:

(5) $$S_{1}=\left\langle \cos\beta_{i}\right\rangle$$

The polar order parameter $S_{1}$ is unequal to 0 if a phase is in fact polar. For a non‐polar phase $S_{1}=0$ .

**Figure 4:**
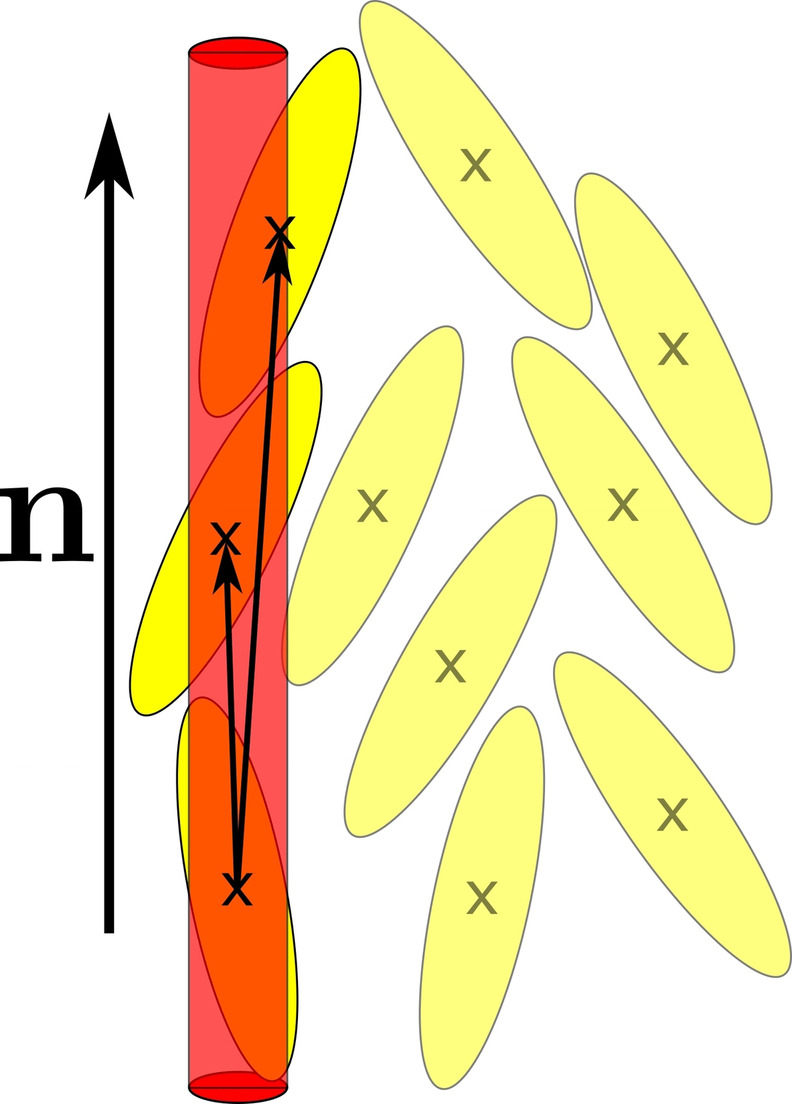
Schematic of one of the constructed cylinders (red) for calculation of the directional pair correlation functions (dPCFs). In this case the calculation direction $h_{\mathrm{cy}}$ is parallel to the director n . The long axis of the cylinder is in the direction of $h_{\mathrm{cy}}$ and through the centres of one particle. The dPCF is increased by 1 at the (projected) distances, for which both particles are within the constructed cylinder. This procedure is repeated for each particle.

Directional pair correlation functions (dPCFs), sometimes also called cylindrical pair correlation functions, quantify the probability to find the centre of mass of a mesogen at a distance r from the centre of a reference particle.^[23]^ They are calculated in the directions parallel ($g\left(r_{\left|\right|}\right)$ ) or perpendicular ($g\left(r_{⊥}\right)$ ) to the director or, in the case of isotropic phases, in an arbitrary direction ($g\left(r\right)$ ):

First, the calculation direction $h_{\mathrm{cy}}$ is set to be along or perpendicular to the director or, in the case of isotropic phases, to an arbitrary direction. Starting from one particle the distance to a second particle projected onto $h_{\mathrm{cy}}$ is determined. The function is increased by 1 at this (projected) distance if both particle centres are within a cylinder which has its long axis in direction of $h_{\mathrm{cy}}$ and passes through the first particles centre. The reduced radius of the cylinder is 0.5, which is the same as the reduced radius of the short axis of the particles. The construction of the cylinder for one particle is sketched in Figure 4. This procedure is repeated for each particle and the function is normalized. For an isotropic phase the dPCFs give the same result as radial distribution functions.

**Figure 5:**
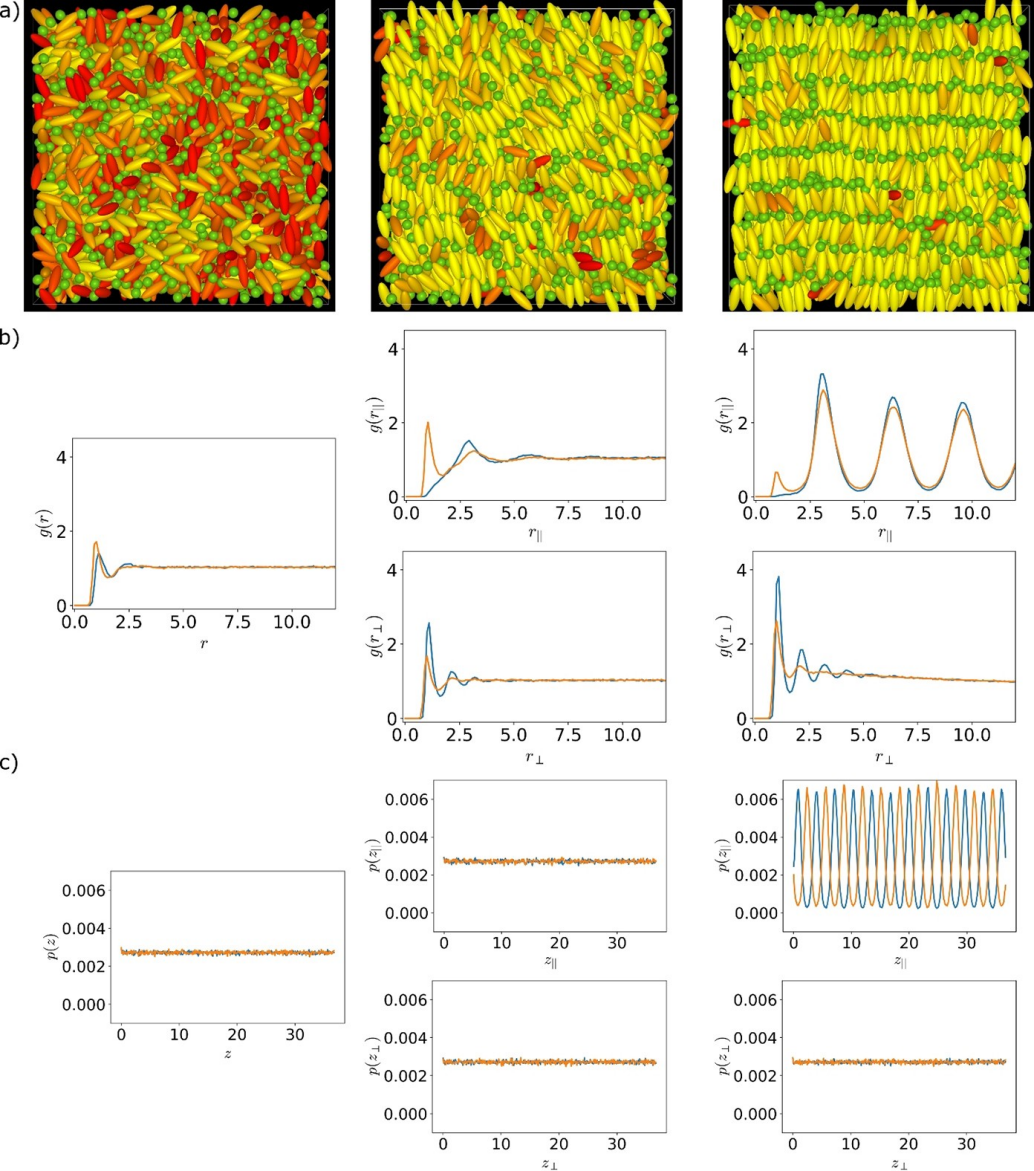
a) Simulation snapshots, b) directional pair correlation functions and c) directional density‐distributions distributions of an isotropic (left), a nematic (middle) and a smectic A phase (right). The examples for the isotropic and nematic phase are taken from simulations with the charge in the middle of the Gay‐Berne (GB) particle at the reduced charge position $z_{c}^{{}^{*}}=0.125$ and reduced temperature $T^{{}^{*}}=3.3$ and $T^{{}^{*}}=2.2$ respectively. The example for a smectic phase is taken from simulations with the charge at the tip of a GB particle at $z_{c}^{{}^{*}}=0.8$ and $T^{{}^{*}}=2.75$ . a) A picture of the 1,000,000^th^ simulation snapshot of the set reduced temperature. The LJ particles are drawn in green. The colour code of the GB particles is according to their orientation to the director n . A particle with a high angle β between n and its long axis appears redder, while particles that have a small β are more yellow. The order parameters of the snapshots GB particles are $S_{2}=0.0$ , Σ=0.0 (left), $S_{2}=0.64$ , Σ=0.0 (middle) and $S_{2}=0.79$ , Σ=0.48 (right). b) Pair correlation functions and c) probability distributions calculated for GB (blue) and the Lennard‐Jones particles (orange). For the isotropic phase the direction in which the function or distribution is calculated is arbitrary. For the nematic and smectic A phase the top distribution is calculated parallel to the director, while the bottom one is calculated orthogonal to the director.

Further analytic tools are directional density distribution functions $p\left(z\right)$ , that describe the probability of finding a molecular centre at a certain z , where z is the centre position projected onto a certain direction. We calculate distributions along ($p\left(z_{\left|\right|}\right)$ ) and orthogonal ($p\left(z_{⊥}\right)$ ) to the director or in the case of an isotropic phase, along an arbitrary ($p\left(z\right)$ ) direction. For a given reduced temperature all order parameters, dPCFs and probability functions are obtained by averaging the results of 100 snapshots (every 1000^th^ snapshot in the range of snapshots 901,000 to 1,000,000).

## Results and Discussion

Multiple factors are considered to assign the molecular arrangement in simulation snapshots to a certain liquid crystalline or isotropic phase: The orientational order parameter $S_{2}$ , the translational order parameter Σ , directional pair correlation functions $g\left(r_{\left|\right|}\right)$ and$\;g\left(r_{⊥}\right)$ or the isotropic $g\left(r\right)$ , directional density distribution functions $p\left(z_{\left|\right|}\right)$ and$\;p\left(z_{⊥}\right)$ or the isotropic $p\left(z\right)$ and pictures of the simulation snapshot themselves.

Visual inspection of the snapshots already gives a good idea about the nature of the underlying phase: A snapshot of an isotropic phase shows no common direction of orientational ordering; a director is however clearly visible in nematic and smectic snapshots. In case of a SmA phase, layers can be easily spotted. Examples for simulation snapshots can be seen in Figure 5a.

The visual inspection of the snapshots in Figure 5a is complemented by the corresponding dPCFs in Figure 5b. As expected, the $g\left(r\right)$ in the isotropic phase, $g\left(r_{⊥}\right)$ and $g\left(r_{\left|\right|}\right)$ in the nematic phase as well as $g\left(r_{⊥}\right)$ in the SmA phase all show the rapid decay characteristic of fluid short range order. The characteristic period of these functions corresponds to the respective molecular dimensions. The correlation lengths slightly increase from the isotropic to the SmA phases. In agreement with experimental results,^[24]^ the presence of quasi long‐range 1D‐translational order is clearly displayed by the weak algebraic decay of $g\left(r_{\left|\right|}\right)$ in SmA.

Other tools for identifying the phases are the density correlation functions $p\left(z\right)$ , $p\left(z_{\left|\right|}\right)$ and $p\left(z_{⊥}\right)$ . The examples in Figure 5c show, that the isotropic and nematic phases have randomly distributed centres indicating short range fluid order. For the smectic phase this is also true orthogonal to the director, confirming the presence of fluid intra‐layer order of a smectic A phase. Only the distribution function $p\left(z_{\left|\right|}\right)$ parallel to the director shows a clear density wave and thus confirms the long range 1D‐translational order in SmA. This analysis was done for all simulation temperatures. The simulation energies, snapshots, order parameters, dPCFs and directional density distribution functions for selected temperatures can be found in the SI.

Even though the mesogenic ions are strongly polar for charge positions $z_{c}^{{}^{*}}>0$ , the resulting phases are non‐polar as is shown by the polar order parameter $S_{1}\;$ (see SI).

We also note that the smectic A phases we simulated are all monolayered and not bilayered as observed for ILCs in experiments. To the best of our knowledge there have only ever been monolayered structures reported in literature[^10^, ^11^, ^25^] for simulated ILCs when using the Gay‐Berne potential. While atomistic and less coarse simulations of ILCs lead to bilayered structures,^[26]^ the GB potential is too simple to realistically describe the specific molecular interactions between flexible hydrocarbon chains necessary for the formation of double layers in ILCs.

**Figure 6:**
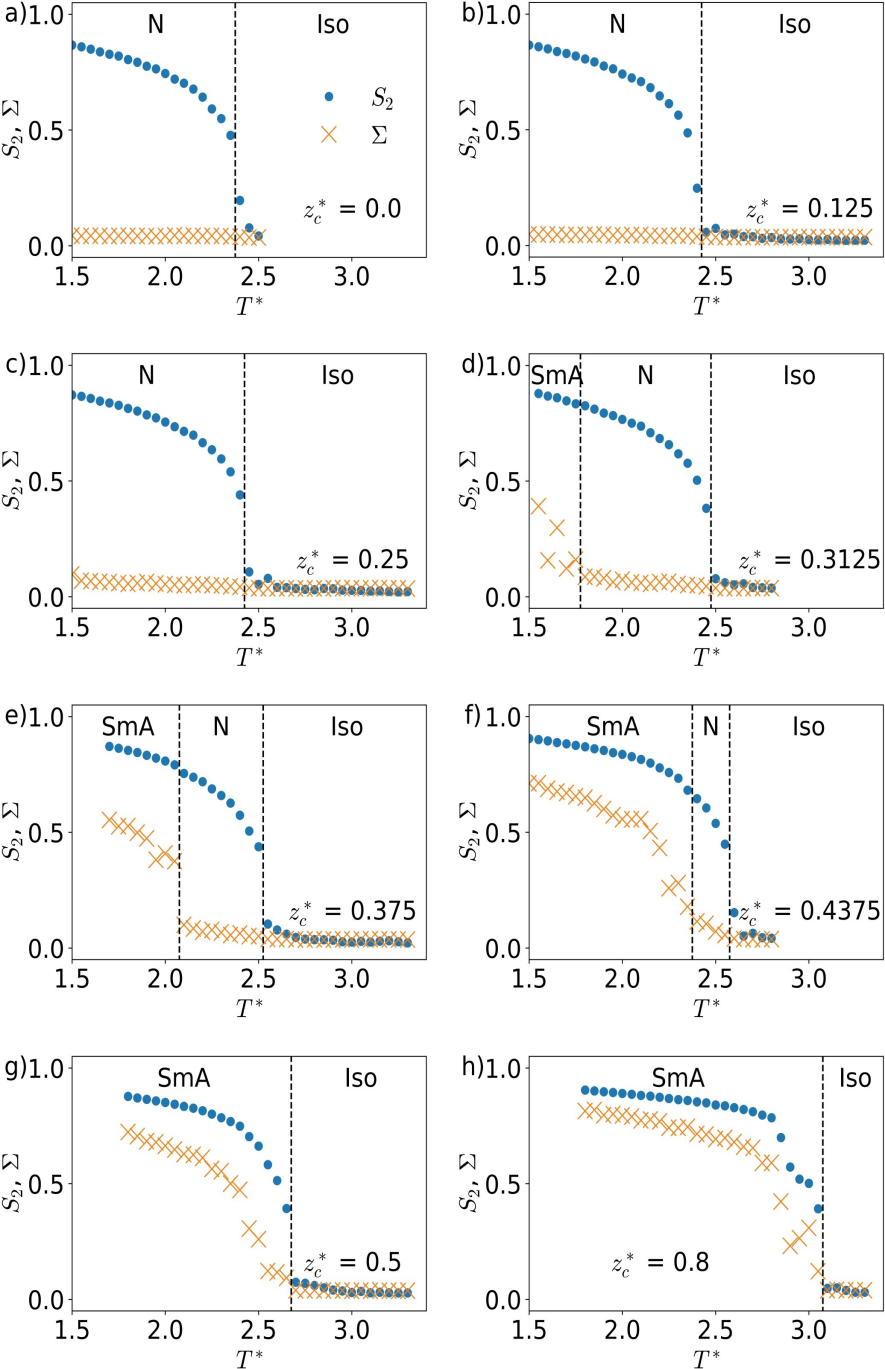
Orientational order parameter (blue circles) and translational order parameter (orange crosses) of the Gay‐Berne particles plotted vs. the reduced temperature $T^{{}^{*}}$ for different charge positions $z_{c}^{{}^{*}}$ . a) $z_{c}^{{}^{*}}=0.0$ b) $z_{c}^{{}^{*}}=0.125$ c) $z_{c}^{{}^{*}}=0.25$ d) $z_{c}^{{}^{*}}=0.3125$ e) $z_{c}^{{}^{*}}=0.375\;f)$ $z_{c}^{{}^{*}}=0.4375$ g) $z_{c}^{{}^{*}}=0.5$ h) $z_{c}^{{}^{*}}=0.8$ .

To get an overview of the phases actually observed in the simulations, the order parameters and energies are plotted vs. the temperature (Figure 6). As seen in Figure 6a the simulations with the charge located right at the centre of the mesogenic ion show a nematic phase below $T^{{}^{*}}=2.35$ . In comparison with the result by Saielli et al.[^10^, ^11^] this transition is around ${\Delta T}^{{}^{*}}=\;0.6$ higher. The transition into the smectic crystalline phase (see SI) is around $T^{{}^{*}}=0.95$ and therefore at slightly lower temperatures than the same transition in Saielli et al.[^10^, ^11^] simulations. These differences originate from our treatment of the mixed interaction by mixing rules.

As seen in Figure 6h (and in the SI), the simulation with the charge at the tip of the GB particle ($z_{c}^{{}^{*}}=0.8$ ) leads to a SmA phase at reduced temperatures below $T^{{}^{*}}=3.05$ . In the simulation with $z_{c}^{{}^{*}}=0.5$ (Figure 6g) the phase transition to SmA is shifted to lower temperatures ($T^{{}^{*}}\leq 2.65$ ). This indicates that the SmA phase is destabilized upon moving the charge of a mesogen closer to its centre. Moving the charge even further to the centre, a nematic state appears at temperatures above SmA. Finally, for charge positions very close to the centre, the segregation tendency becomes so small that SmA phases disappear, and only nematic phases are observed. All phase sequences are listed in Table 1.

**Table 1:** Transition temperatures for simulations with different charge positions $z_{c}^{{}^{*}}$ (Isotropic Phase: Iso, Nematic Phase: N, Smectic A phase: SmA).

Reduced charge position $z_{c}^{{}^{*}}$	Transitions, $T^{{}^{*}}$
0	Iso 2.35 N
0.125	Iso 2.4 N
0.25	Iso 2.4 N 1.35 SmA
0.3125	Iso 2.45 N 1.75 SmA
0.375	Iso 2.5 N 2.05 SmA
0.4375	Iso 2.55 N 2.35 SmA
0.5	Iso 2.65 SmA
0.8	Iso 3.05 SmA

**Figure 7:**
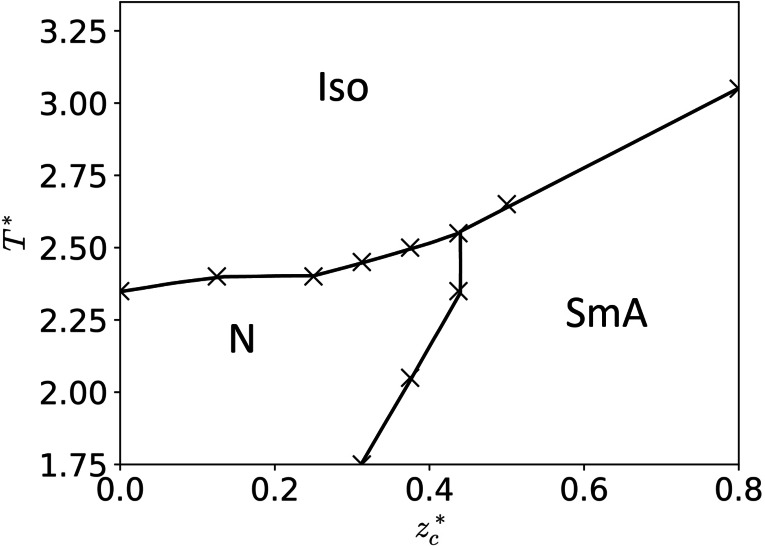
Phase diagram depending on the reduced charge position $z_{c}^{{}^{*}}$ . The smectic A phase (SmA) is favoured for charge positions starting at roughly 0.4 the distance between the centre and the tip of the GB particle. Only when the charge is located close to the centre of the GB particle the nematic phase (N) occurs. Iso: Isotropic.

These findings are summarized in the phase diagram in Figure 7 essentially showing three regimes of mesomorphism: (i) If the charge is located close to the end of the mesogen the segregation between ionic and non‐ionic parts is easily possible and thus SmA is the only liquid crystal phase observed. (ii) If the charge is located close to the centre of the mesogen the segregation between ionic and non‐ionic parts is hardly possible and thus a nematic phase instead of a SmA phase is found. (iii) In the intermediate regime both phases are actually observed.

Our simulations show a coherent picture how the phase behaviour of ILCs changes if the charge is moved from the tip to the centre of the ionic mesogen. The theoretical results of Kondrat et al.^[27]^ as well as the experimental results of Pană et al.[^6^, ^7^] perfectly fit into this picture.

Coming back to our hypothesis, that the location of charge on a mesogenic ion is one of the keys to nematic ILCs, it becomes clear that the hypothesis holds true: The nematic state is stabilized, and the smectic state is destabilized when the charge is at or close to the centre of the mesogenic ion and vice versa.

## Conclusions

In this paper we present simulations of mixtures of charged GB and LJ particles, where the position of charge on the GB particle is varied between simulation runs. These simulations let us investigate the phase behaviour of an ILCs in dependence of its charge position, more specifically we investigate how the position of the charge affects the stability of nematic or SmA phases.

We find that the nematic phase only occurs if the charge on the mesogenic unit is located close to the centre of the mesogenic ion. All in all, one can say that shifting the charge from the end towards the centre of the mesogenic ion destabilizes the liquid crystalline state with respect to the isotropic state and induces a change from SmA to nematic phases.

These results suggest that experimental attempts to find nematic ILCs should focus on centring the ionic charge in the middle of the mesogenic unit. Further parameters which might be important for the stability of nematic ILCs are the polarizabilities of both the mesogenic and the counter ion, as well as the relative size of the counter ion, which might be investigated by further simulation efforts.

## Conflict of interest

The authors declare no conflict of interest.

1

## Supporting information

As a service to our authors and readers, this journal provides supporting information supplied by the authors. Such materials are peer reviewed and may be re‐organized for online delivery, but are not copy‐edited or typeset. Technical support issues arising from supporting information (other than missing files) should be addressed to the authors.

Supporting Information

## Data Availability

The data that support the findings of this study are available in the supplementary material of this article.
