# Supplementary material for: Towards Nematic Phases in Ionic Liquid Crystals – A Simulation Study
Source: Chemphyschem. 2022 Oct 25;24(1):e202200424. doi: 10.1002/cphc.202200424 (PMC10092135; doi:10.1002/cphc.202200424)
Supplement: Supplementary file 1 — Supporting Information [file CPHC-24-0-s001.pdf]

ChemPhysChem

Supporting Information

Towards Nematic Phases in Ionic Liquid Crystals – A Simulation Study

Christian Haege, Stefan Jagiella, and Frank Giesselmann*

Table of contents

Gay-Berne potential 2

Simulation details 2

Simulation runs 3

Simulation results 3

References 30

Gay-Berne potential

The Gay-Berne[1] (GB) potential $U(\hat{\mathbf{u}}_1,\hat{\mathbf{u}}_2,\mathbf{r})$ is used to simulate anisotropic particles. The following parameters have to be set in ESPResSo[2]: The ratio of the diameters of the semi-major and semi-minor axes k_1, the ratio of the potentials side-by-side and end-to-end configurations well depths k_2, the diameter of the semi-minor axis σ_0, the well depth of the end-to-end configuration ϵ_0 and the adjustable exponents μ and ν. The GB potential $U(\hat{\mathbf{u}}_1,\hat{\mathbf{u}}_2,\mathbf{r})$ is given by

$$U(\hat{\mathbf{u}}_1,\hat{\mathbf{u}}_2,\mathbf{r}) = 4\epsilon(\hat{\mathbf{u}}_1,\hat{\mathbf{u}}_2,\hat{\mathbf{r}})\left[\left(\frac{\sigma_0}{r-\sigma(\hat{\mathbf{u}}_1,\hat{\mathbf{u}}_2,\hat{\mathbf{r}})+\sigma_0}\right)^{12} - \left(\frac{\sigma_0}{r-\sigma(\hat{\mathbf{u}}_1,\hat{\mathbf{u}}_2,\hat{\mathbf{r}})+\sigma_0}\right)^{6}\right]. \tag{S1}$$

Here $\mathbf{r} = |\mathbf{r}|\hat{\mathbf{r}}$ is the vector between the two interactions sites, $\hat{\mathbf{u}}_1$ and $\hat{\mathbf{u}}_2$ are unit vectors describing the orientation of the interaction sites. The spatial extent of the interaction sites is orientation deepened and is given by

$$\sigma(\hat{\mathbf{u}}_1,\hat{\mathbf{u}}_2,\hat{\mathbf{r}}) = \sigma_0\left(1-\frac{\chi}{2}\left[\frac{(\hat{\mathbf{r}}\cdot\hat{\mathbf{u}}_1+\hat{\mathbf{r}}\cdot\hat{\mathbf{u}}_2)^2}{1+\chi(\hat{\mathbf{u}}_1\cdot\hat{\mathbf{u}}_2)} + \frac{(\hat{\mathbf{r}}\cdot\hat{\mathbf{u}}_1-\hat{\mathbf{r}}\cdot\hat{\mathbf{u}}_2)^2}{1-\chi(\hat{\mathbf{u}}_1\cdot\hat{\mathbf{u}}_2)}\right]\right)^{-1/2}. \tag{S2}$$

The well depth is also orientation depended. It is given by

$$\epsilon(\hat{\mathbf{u}}_1,\hat{\mathbf{u}}_2,\hat{\mathbf{r}}) = \epsilon^{\nu}(\hat{\mathbf{u}}_1,\hat{\mathbf{u}}_2)\cdot\epsilon'^{\mu}(\hat{\mathbf{u}}_1,\hat{\mathbf{u}}_2,\hat{\mathbf{r}}). \tag{S3}$$

Here

$$\epsilon(\hat{\mathbf{u}}_1,\hat{\mathbf{u}}_2) = \epsilon_0^{1/\nu}[1-\chi^2(\hat{\mathbf{u}}_1\cdot\hat{\mathbf{u}}_2)^2]^{-1/2} \tag{S4}$$

and

$$\epsilon'(\hat{\mathbf{u}}_1,\hat{\mathbf{u}}_2,\hat{\mathbf{r}}) = 1-\frac{\chi'}{2}\left[\frac{(\hat{\mathbf{r}}\cdot\hat{\mathbf{u}}_1+\hat{\mathbf{r}}\cdot\hat{\mathbf{u}}_2)^2}{1+\chi'(\hat{\mathbf{u}}_1\cdot\hat{\mathbf{u}}_2)} + \frac{(\hat{\mathbf{r}}\cdot\hat{\mathbf{u}}_1-\hat{\mathbf{r}}\cdot\hat{\mathbf{u}}_2)^2}{1-\chi'(\hat{\mathbf{u}}_1\cdot\hat{\mathbf{u}}_2)}\right]. \tag{S5}$$

The parameters χ and χ' are given by

$$\chi = \frac{k_1^2-1}{k_1^2+1} \tag{S6}$$

and

$$\chi' = \frac{k_2^{1/\mu}-1}{k_2^{1/\mu}+1}. \tag{S7}$$

Simulation details

The Coulomb interaction is calculated using a particle-particle-particle-mesh method[2,3], with the accuracy set to $1\cdot 10^{-6}$. The Coulomb interaction is given by

$$E_C = C\cdot\frac{q_1^* q_2^*}{r}. \tag{S8}$$

Here $C = 1$ is a prefactor, q_1^* and q_2^* are reduced charges and r is the distance between the two charges.

Apart from the coulomb interaction, three non-bonded interactions are defined: The Gay-Berne (GB) to GB interaction, the Lennard-Jones (LJ) to LJ interaction and the GB to LJ interaction. The parameters of the GB interaction are equal to the ones used by Beradi et al.[4] and Saielli et al.[5]. The parameters of the LJ interaction, the Coulomb interaction, the number density and the number of particles are taken from Saielli et al.[5]. The GB-to-GB interaction is modelled using the parameters $k_1 = 3, k_2 = 5, \mu = 1, \nu = 3, \sigma_0 = 1, \epsilon_0 = 1$. The LJ-to-LJ interaction uses parameters $\sigma = 1$ and $\epsilon = 1$. Here σ is the diameter of the LJ particle and ϵ is the potentials well depth. The LJ potential is the classic 12-6 potential. The parameters of the interaction between the GB and the LJ particles is calculated by Lorentz[6] and Berthelot[7] mixing rules[8]. It is therefore modelled like a GB interaction with parameters

$k_1 = 2$, $k_2 = \sqrt{5}$, $\mu = 1$, $\nu = 3$, $\sigma_0 = 1$, $\epsilon_0 = 1$. We note that this has one important implication for the visual display of snapshots. GB and LJ particles can look like they are overlapping in a snapshot, while they are in fact not overlapping in a physical sense (see Figure S1).

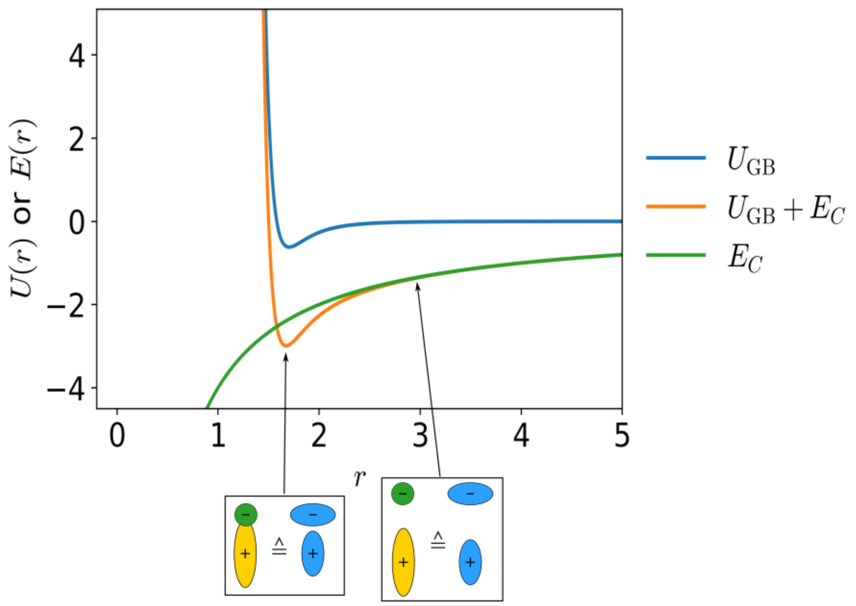

Figure S1: The mixed interaction between the spherical LJ particles (green) and the prolate GB particles (yellow) is approximated according to the mixing rule by the side-to-end Gay-Berne interaction of the blue ellipsoids, which leads to the blue curve $U_{\rm GB}(r)$. The Coulomb interaction between the particles of opposite charge is shown by the green curve $E_C(r)$, the sum of both is the total interaction potential shown by the orange curve. As can be seen, the use of the mixed interaction leads to an energy minimum which shows a partial overlap of the GB and the LJ particles, this overlap does not affect the energies as the potentials represented by the green and yellow shapes are not used for calculating the mixed interaction, the relative proportions are the same as in our simulations.

Simulation runs

Every simulation has three parts: Setup, Warmup and the actual simulation runs:

During Setup the interactions are defined, the particles are randomly placed in the simulation box and the Coulomb interaction is tuned. The Coulomb interaction must be tuned using the target box size of $V^* = 24224$. After this the box size is increased by several orders of magnitude. The increase of the box size must be done, otherwise the particles might be too close together and the simulation would "explode".

Warmup consists of 200000 molecular dynamic (md) steps during which the box size is incrementally decreased until the target box size is reached. When the target box size is reached, the Coulomb interaction is retuned.

The actual simulation consists of 1000000 md steps for every temperature. The starting temperature is selected, so that the system starts from an isotropic phase. For most systems the starting temperature is $T^* = 3.3$. The temperature decrease is $\Delta T^* = 0.05$ for all simulations.

Simulation results

Explanation of variables and colour codes

In the following $E_{\rm tot}$ is the total energy, $E_{\rm kin}$ is the total kinetic energy, $E_{\rm nonbonded}$ is the total energy from the interactions between the GB and GB, LJ and LJ, GB and LJ particles and $E_{\rm coulomb}$ is the total coulomb energy. All energies are divided by the number of particles $N_{\rm particles}$. The different functions and order parameters are described in the main text of the paper. All order parameters and functions are averaged over every 1000$^{\rm th}$ snapshot from snapshot 901000-1000000 of the given reduced temperature, giving the results from 100 snapshots averaged. In snapshot pictures the LJ particles are drawn in green. The colour code of the GB particles is according to their

orientation to the director $\mathbf{n}$. A particle with a high angle β between $\mathbf{n}$ and its long axis appears redder, while particles that have a small β are more yellow. If no legend is given for directional pair correlation functions and directional density correlation functions the ones in blue correspond to the GB particles and the ones in orange correspond to the LJ particles.

System with reduced charge position $z_c^* = 0.8$ – overview

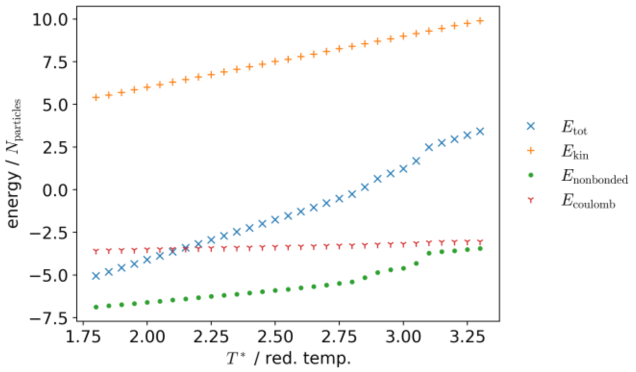

Figure S2: Energies over reduced temperature T^* for the system with reduced charge position $z_c^* = 0.8$.

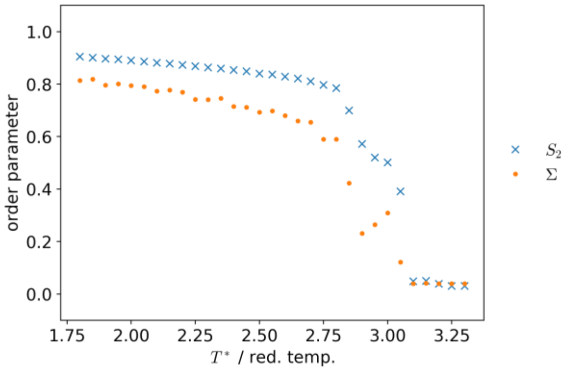

Figure S3: Orientational order parameter S_2 and translational order parameter Σ over reduced temperature T^* for the system with reduced charge position $z_c^* = 0.8$.

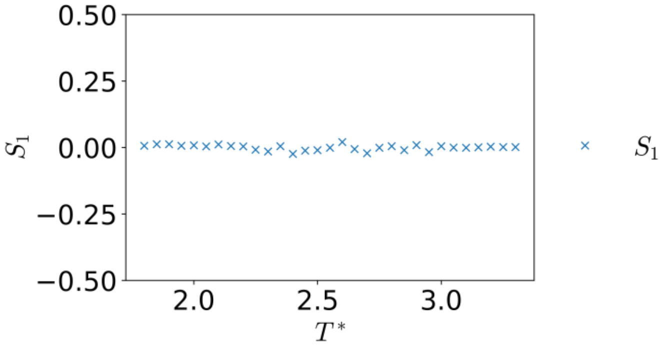

Figure S4: Polar order parameter S_1 over reduced temperature T^* for the system with reduced charge position $z_c^* = 0.8$.

System with reduced charge position $\boldsymbol{z_c^*} = \mathbf{0.8}$ at $\boldsymbol{T^*} = \mathbf{3.3}$ – isotropic phase

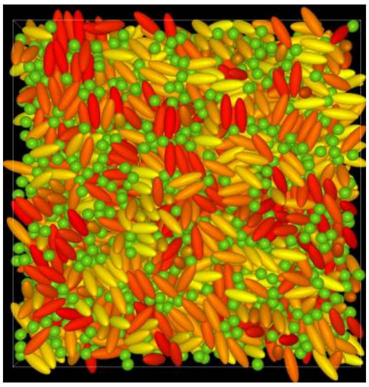

Figure S5: A picture of the 1000000th simulation snapshot at reduced temperature $T^* =$ 3.3 for the system with reduced charge position $z_c^* = 0.8$. The system is in the isotropic phase.

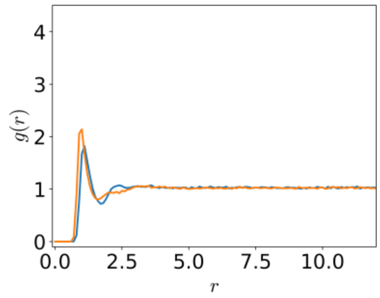

Figure S6: Directional pair correlation functions at reduced temperature $T^* =$ 3.3 for the system with reduced charge position $z_c^* = 0.8$.

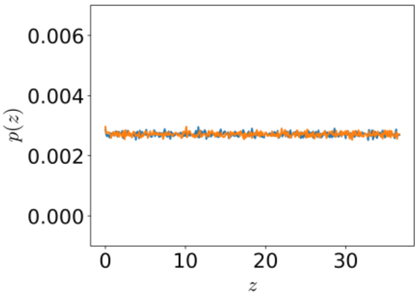

Figure S7: Directional density-distributions at reduced temperature $T^* =$ 3.3 for the system with reduced charge position $z_c^* = 0.8$.

System with reduced charge position $\boldsymbol{z_c^*} = \mathbf{0.8}$ at $\boldsymbol{T^*} = \mathbf{2.95}$ – smectic A phase

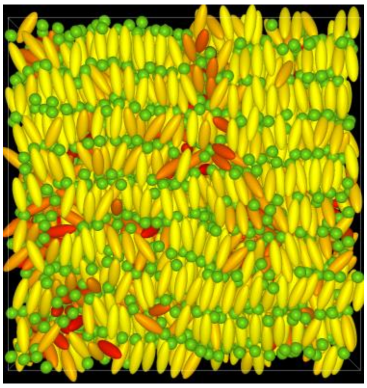

Figure S8: A picture of the 1000000th simulation snapshot at reduced temperature $T^* =$ 2.95 for the system with reduced charge position $z_c^* = 0.8$. The system is in the smectic A phase.

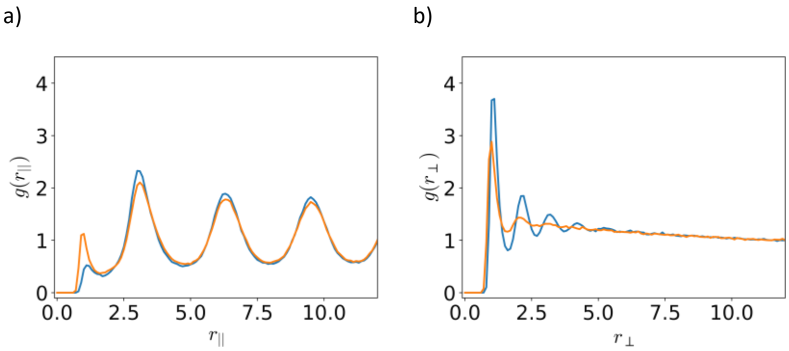

Figure S9: Directional pair correlation functions at reduced temperature $T^* =$ 2.95 for the system with reduced charge position $z_c^* = 0.8$. Calculated parallel (a) and orthogonal (b) to the director of the GB particles.

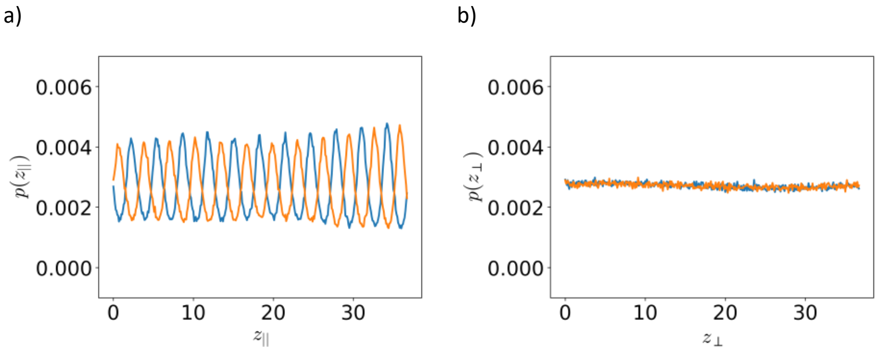

Figure S10: Directional density-distributions at reduced temperature $T^* =$ 2.95 for the system with reduced charge position $z_c^* = 0.8$. Calculated parallel (a) and orthogonal (b) to the director of the GB particles.

System with reduced charge position $z_c^* = 0.5$ – overview

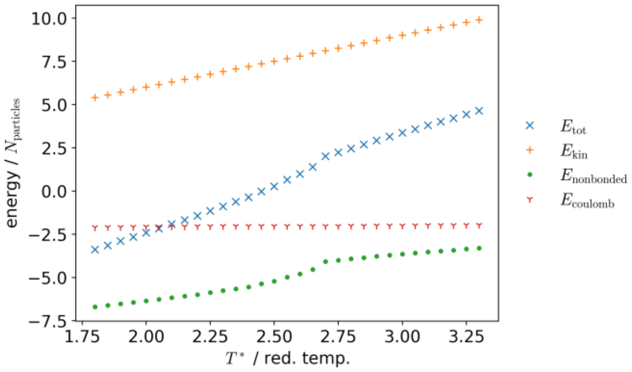

Figure S11: Energies over reduced temperature T^* for the system with reduced charge position $z_c^* = 0.5$.

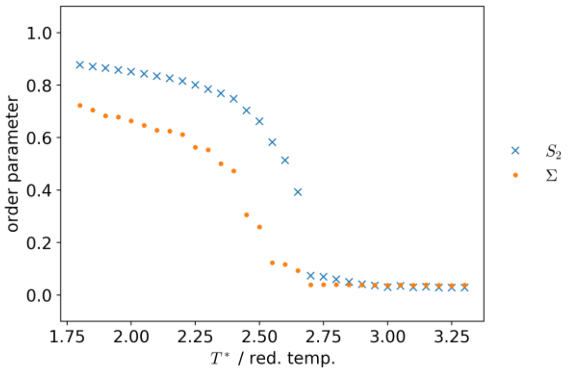

Figure S12: Orientational order parameter S_2 and translational order parameter Σ over reduced temperature T^* for the system with reduced charge position $z_c^* = 0.5$.

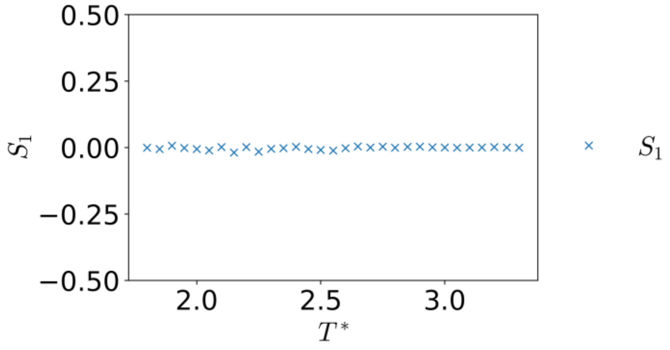

Figure S13: Polar order parameter S_1 over reduced temperature T^* for the system with reduced charge position $z_c^* = 0.5$.

System with reduced charge position $\boldsymbol{z_c^* = 0.5}$ at $\boldsymbol{T^* = 3.3}$ – isotropic phase

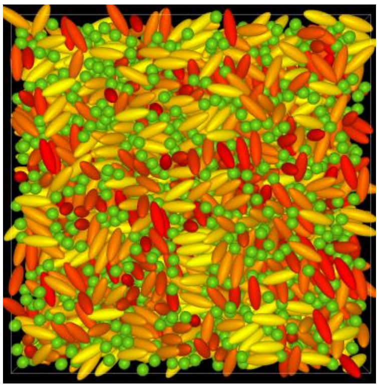

Figure S14: A picture of the 1000000th simulation snapshot at reduced temperature $T^* =$ 3.3 for the system with reduced charge position $z_c^* = 0.5$. The system is in the isotropic phase.

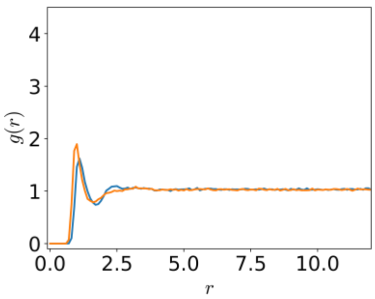

Figure S15: Directional pair correlation functions at reduced temperature $T^* =$ 3.3 for the system with reduced charge position $z_c^* = 0.5$.

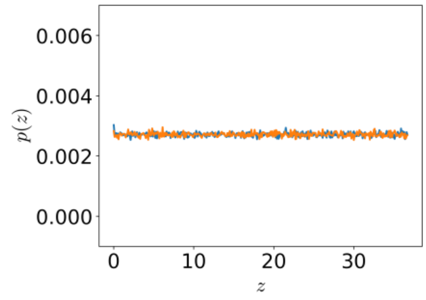

Figure S16: Directional density-distributions at reduced temperature $T^* =$ 3.3 for the system with reduced charge position $z_c^* = 0.5$.

System with reduced charge position $\boldsymbol{z_c^*} = \mathbf{0.5}$ at $\boldsymbol{T^*} = \mathbf{2.5}$ – smectic A phase

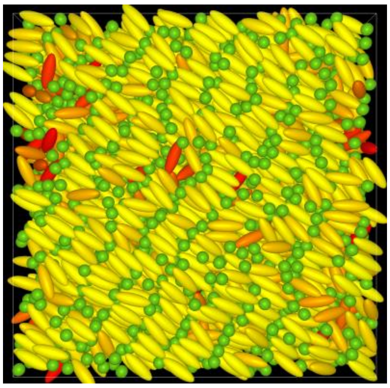

Figure S17: A picture of the 1000000th simulation snapshot at reduced temperature $T^* =$ 2.5 for the system with reduced charge position $z_c^* = 0.5$. The system is in the smectic A phase.

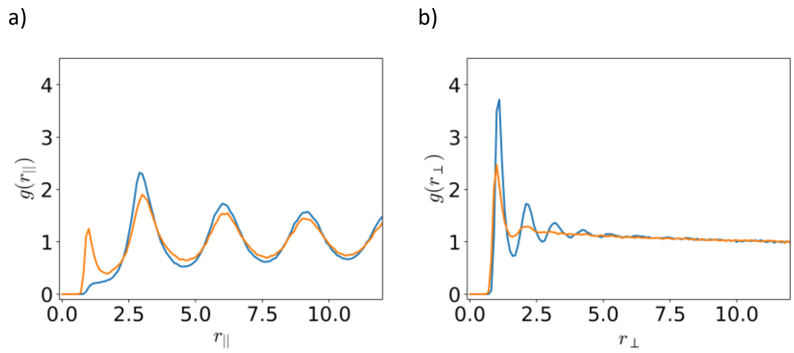

Figure S18: Directional pair correlation functions at reduced temperature $T^* =$ 2.5 for the system with reduced charge position $z_c^* = 0.5$. Calculated parallel (a) and orthogonal (b) to the director of the GB particles.

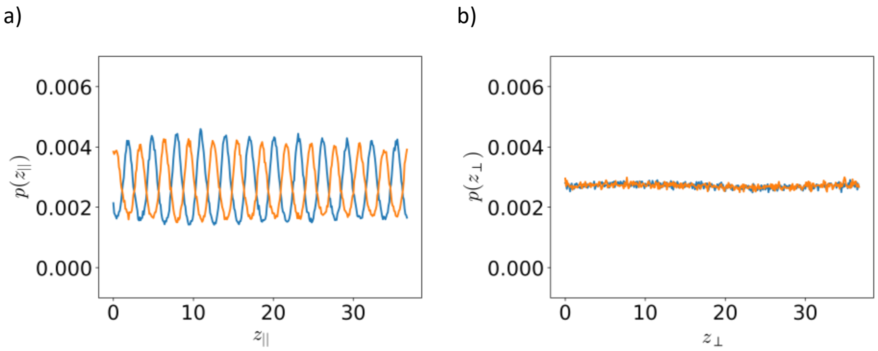

Figure S19: Directional density-distributions at reduced temperature $T^* =$ 2.5 for the system with reduced charge position $z_c^* = 0.5$. Calculated parallel (a) and orthogonal (b) to the director of the GB particles.

System with reduced charge position $z_c^* = 0.4375$ – overview

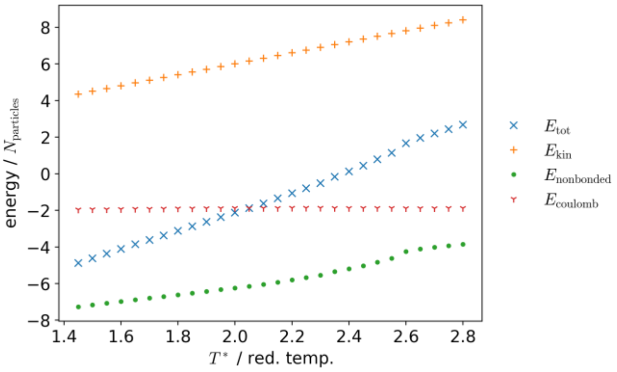

Figure S20: Energies over reduced temperature T^* for the system with reduced charge position $z_c^* = 0.4375$.

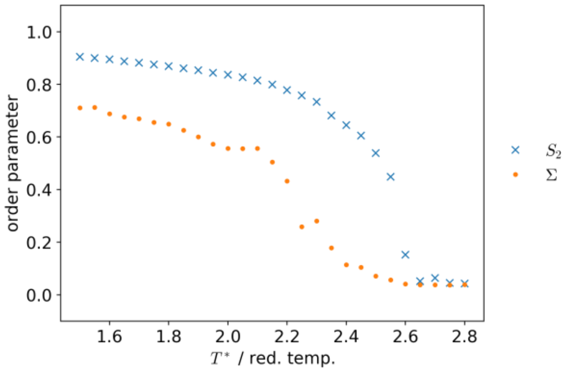

Figure S21: Orientational order parameter S_2 and translational order parameter Σ over reduced temperature T^* for the system with reduced charge position $z_c^* = 0.4375$.

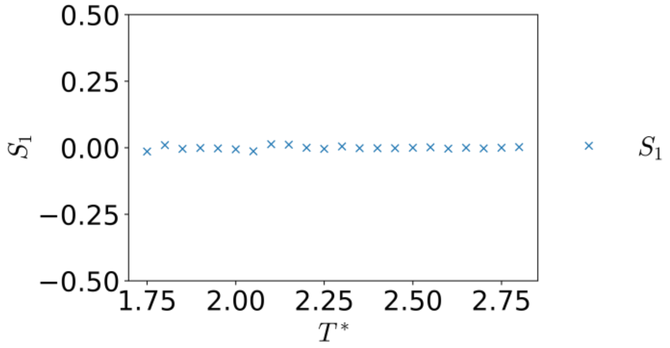

Figure S22: Polar order parameter S_1 over reduced temperature T^* for the system with reduced charge position $z_c^* = 0.4375$.

System with reduced charge position $\boldsymbol{z_c^*} = \mathbf{0.4375}$ at $\boldsymbol{T^*} = \mathbf{2.8}$ – isotropic phase

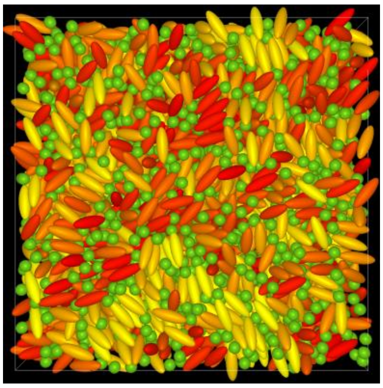

Figure S23: A picture of the 1000000th simulation snapshot at reduced temperature $T^* = 2.8$ for the system with reduced charge position $z_c^* = 0.4375$. The system is in the isotropic phase.

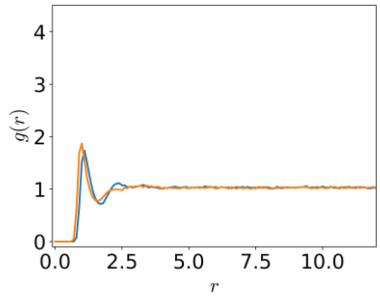

Figure S24: Directional pair correlation functions at reduced temperature $T^* = 2.8$ for the system with reduced charge position $z_c^* = 0.4375$.

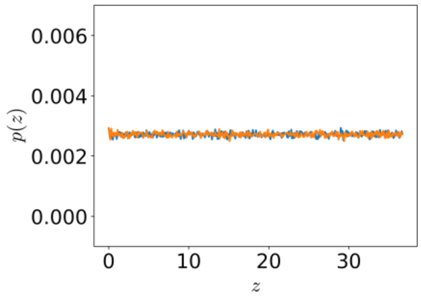

Figure S25: Directional density-distributions at reduced temperature $T^* = 2.8$ for the system with reduced charge position $z_c^* = 0.4375$.

System with reduced charge position $\boldsymbol{z_c^*} = \mathbf{0.4375}$ at $\boldsymbol{T^*} = \mathbf{2.55}$ – nematic phase

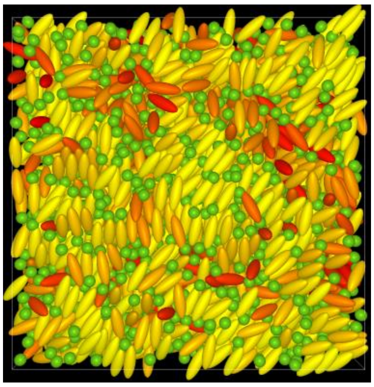

Figure S26: A picture of the 1000000th simulation snapshot at reduced temperature $T^* = 2.55$ for the system with reduced charge position $z_c^* = 0.4375$. The system is in the nematic phase.

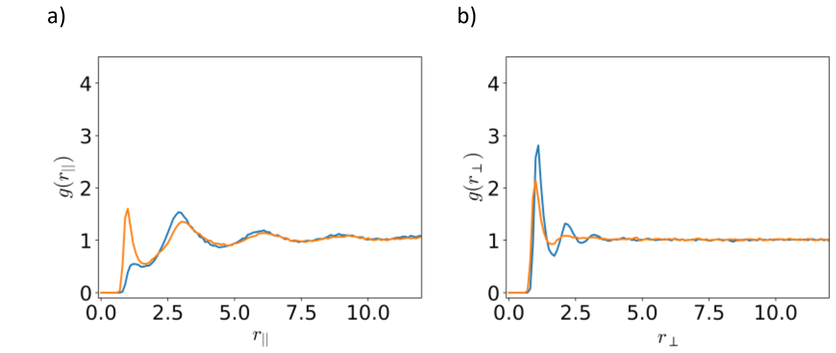

Figure S27: Directional pair correlation functions at reduced temperature $T^* =$ 2.55 for the system with reduced charge position $z_c^* =$ 0.4375. Calculated parallel (a) and orthogonal (b) to the director of the GB particles.

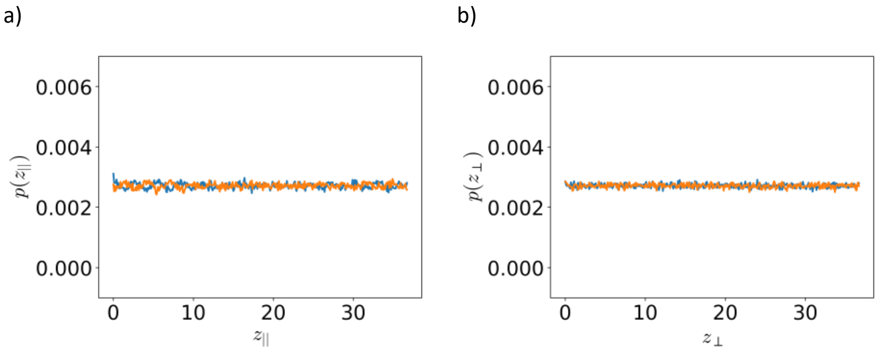

Figure S28: Directional density-distributions at reduced temperature $T^* =$ 2.55 for the system with reduced charge position $z_c^* =$ 0.4375. Calculated parallel (a) and orthogonal (b) to the director of the GB particles.

System with reduced charge position $\boldsymbol{z_c^*} = \mathbf{0.4375}$ at $\boldsymbol{T^*} = \mathbf{2.35}$ – smectic A phase

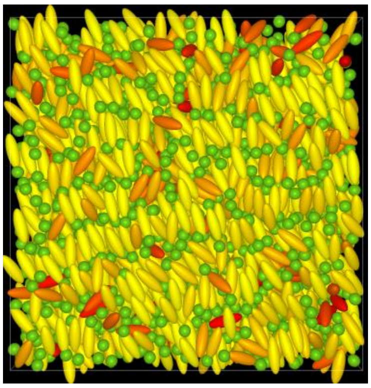

Figure S29: A picture of the 1000000th simulation snapshot at reduced temperature $T^* =$ 2.35 for the system with reduced charge position $z_c^* =$ 0.4375. The system is in the smectic A phase.

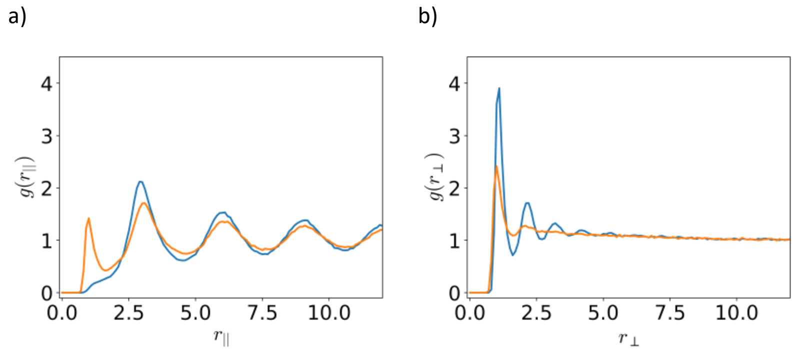

Figure S30: Directional pair correlation functions at reduced temperature $T^* = 2.35$ for the system with reduced charge position $z_c^* = 0.4375$. Calculated parallel (a) and orthogonal (b) to the director of the GB particles.

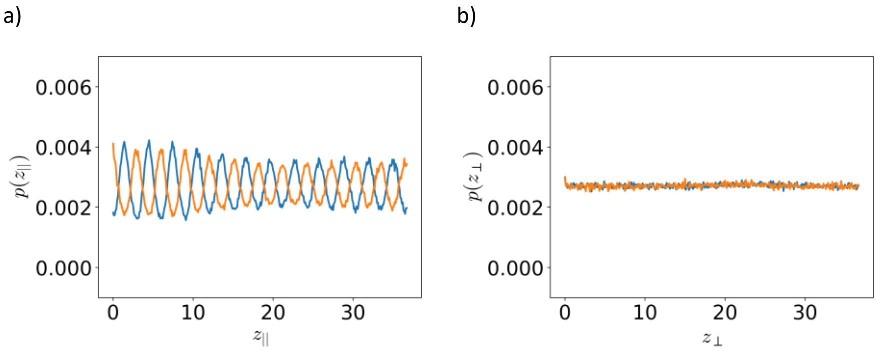

Figure S31: Directional density-distributions at reduced temperature $T^* = 2.35$ for the system with reduced charge position $z_c^* = 0.4375$. Calculated parallel (a) and orthogonal (b) to the director of the GB particles.

System with reduced charge position $\boldsymbol{z_c^*} = \mathbf{0.375}$ – overview

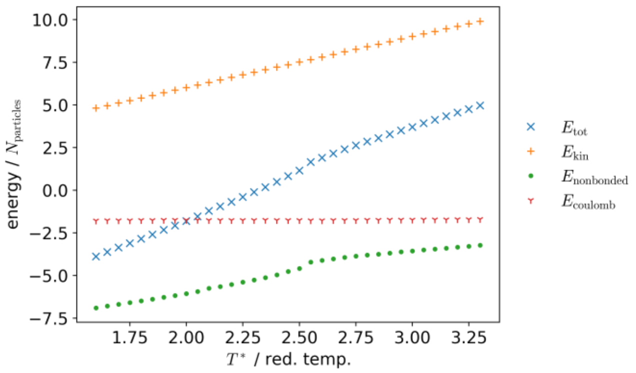

Figure S32: Energies over reduced temperature T^* for the system with reduced charge position $z_c^* = 0.375$.

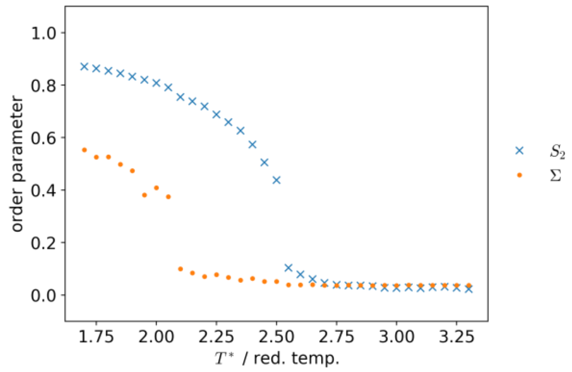

Figure S33: Orientational order parameter S_2 and translational order parameter Σ over reduced temperature T^* for the system with reduced charge position $z_c^* = 0.375$.

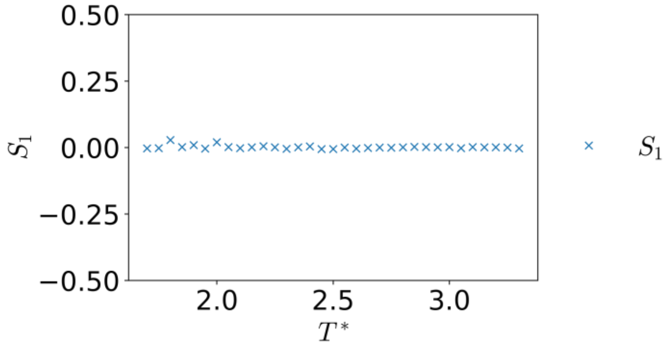

Figure S34: Polar order parameter S_1 over reduced temperature T^* for the system with reduced charge position $z_c^* = 0.375$.

System with reduced charge position $\boldsymbol{z_c^*} = \mathbf{0.375}$ at $\boldsymbol{T^*} = \mathbf{3.3}$ – isotropic phase

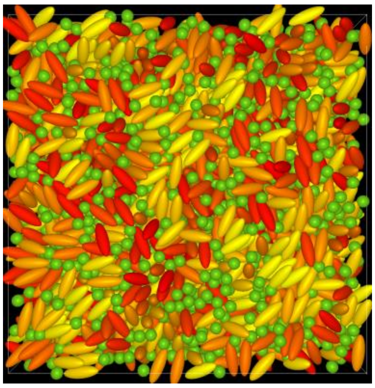

Figure S35: A picture of the 1000000th simulation snapshot at reduced temperature $T^* =$ 3.3 for the system with reduced charge position $z_c^* = 0.375$. The system is in the isotropic phase.

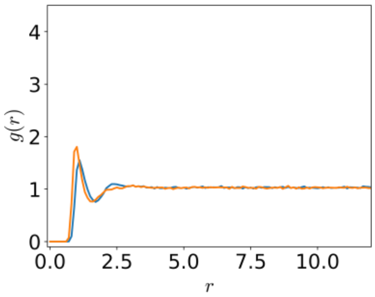

Figure S36: Directional pair correlation functions at reduced temperature $T^* =$ 3.3 for the system with reduced charge position $z_c^* = 0.375$.

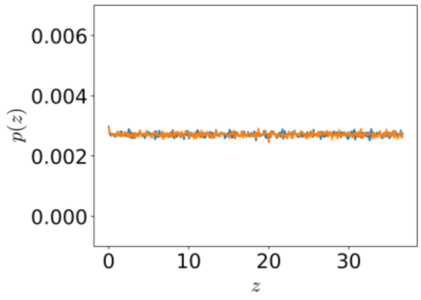

Figure S37: Directional density-distributions at reduced temperature $T^* =$ 3.3 for the system with reduced charge position $z_c^* = 0.375$.

System with reduced charge position $\boldsymbol{z_c^*} = \mathbf{0.375}$ at $\boldsymbol{T^*} = \mathbf{2.45}$ – nematic phase

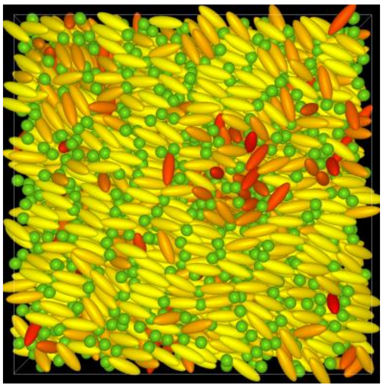

Figure S38: A picture of the 1000000th simulation snapshot at reduced temperature $T^* =$ 2.45 for the system with reduced charge position $z_c^* = 0.375$. The system is in the nematic phase.

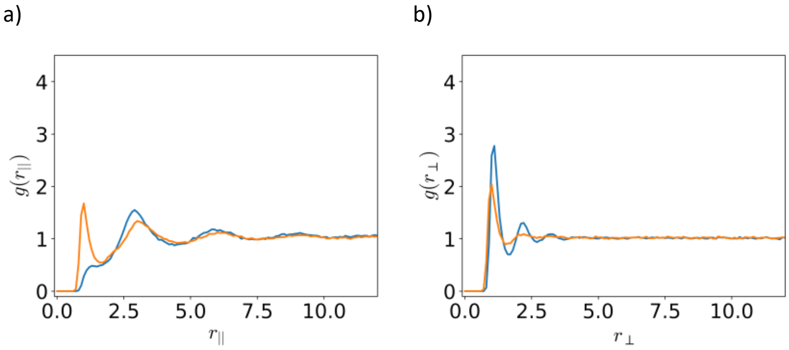

Figure S39: Directional pair correlation functions at reduced temperature $T^* =$ 2.45 for the system with reduced charge position $z_c^* =$ 0.375. Calculated parallel (a) and orthogonal (b) to the director of the GB particles.

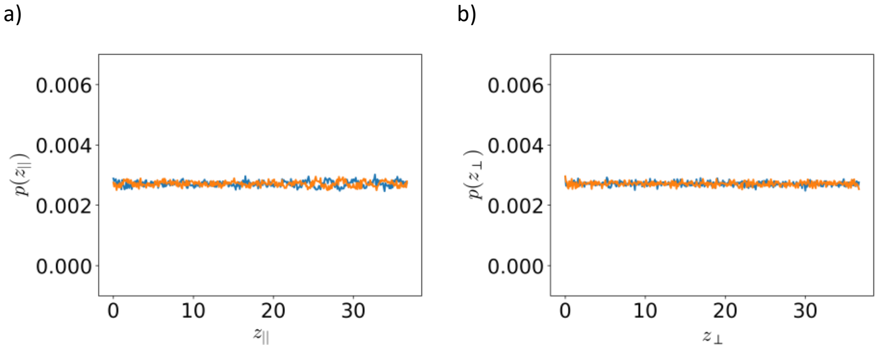

Figure S40: Directional density-distributions at reduced temperature $T^* =$ 2.45 for the system with reduced charge position $z_c^* = 0.375$. Calculated parallel (a) and orthogonal (b) to the director of the GB particles.

System with reduced charge position $z_c^* = 0.375$ at $T^* = 2.0$– smectic A phase

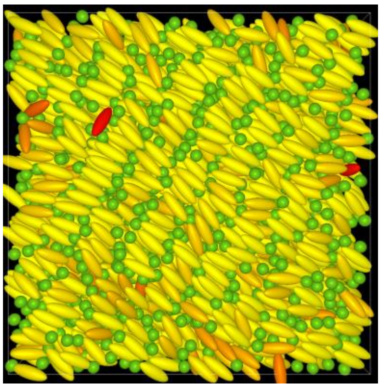

Figure S41: A picture of the 1000000th simulation snapshot at reduced temperature $T^* =$ 2.0 for the system with reduced charge position $z_c^* = 0.375$. The system is in the smectic A phase.

a) b)

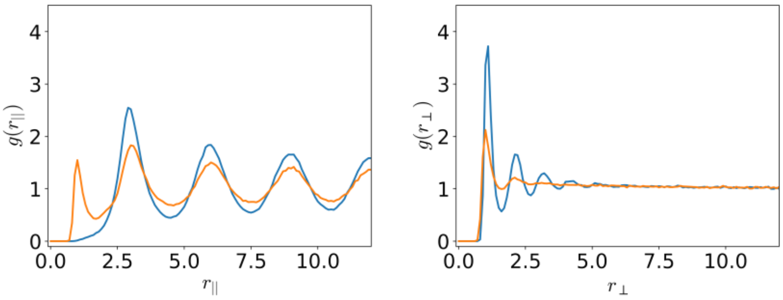

Figure S42: Directional pair correlation functions at reduced temperature $T^* =$ 2.0 for the system with reduced charge position $z_c^* = 0.375$. Calculated parallel (a) and orthogonal (b) to the director of the GB particles.

a) b)

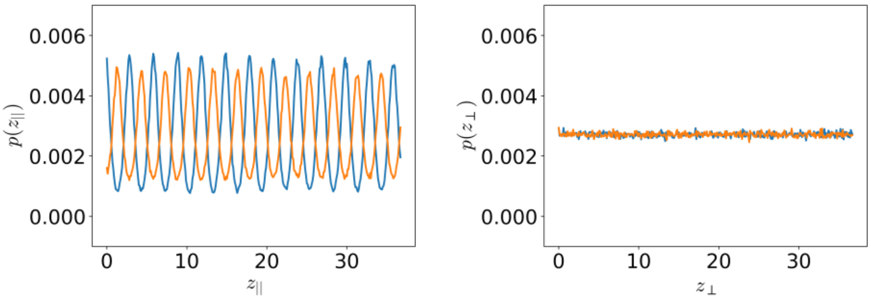

Figure S43: Directional density-distributions at reduced temperature $T^* =$ 2.0 for the system with reduced charge position $z_c^* = 0.375$. Calculated parallel (a) and orthogonal (b) to the director of the GB particles.

System with reduced charge position $z_c^* = 0.3125$ – overview

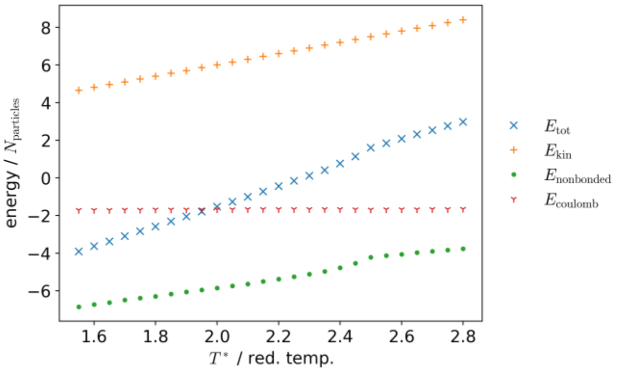

Figure S44: Energies over reduced temperature T^* for the system with reduced charge position $z_c^* = 0.3125$.

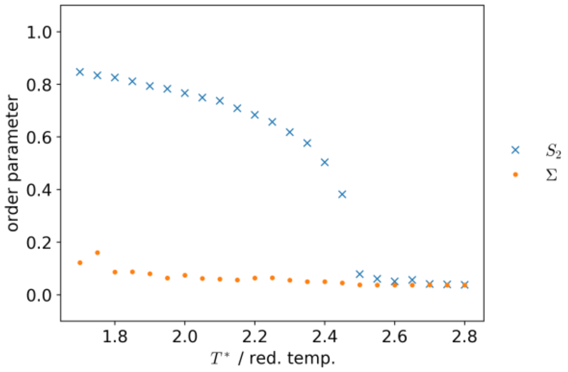

Figure S45: Orientational order parameter S_2 and translational order parameter Σ over reduced temperature T^* for the system with reduced charge position $z_c^* = 0.3125$.

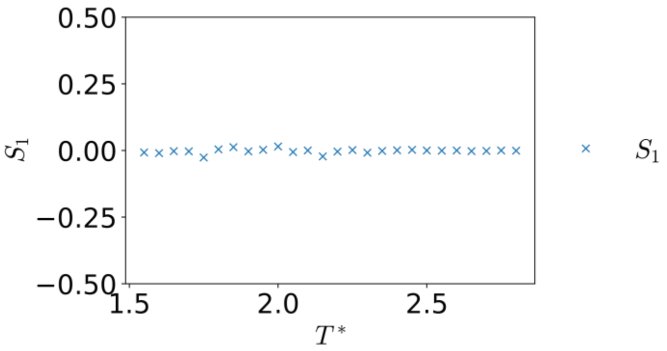

Figure S46: Polar order parameter S_1 over reduced temperature T^* for the system with reduced charge position $z_c^* = 0.3125$.

System with reduced charge position $z_c^* = 0.3125$ at $T^* = 2.8$ – isotropic phase

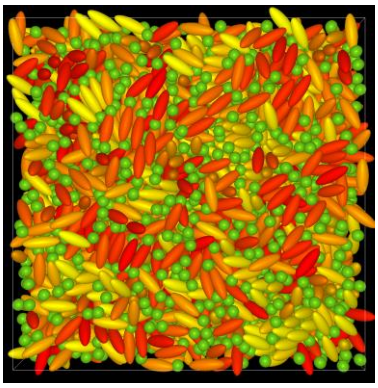

Figure S47: A picture of the 1000000th simulation snapshot at reduced temperature $T^* =$ 2.8 for the system with reduced charge position $z_c^* = 0.3125$. The system is in the isotropic phase.

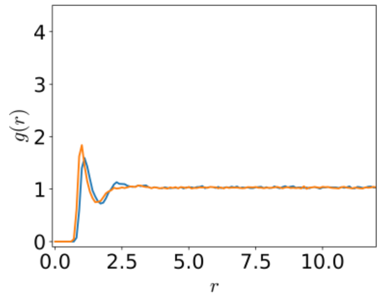

Figure S48: Directional pair correlation functions at reduced temperature $T^* =$ 2.8 for the system with reduced charge position $z_c^* =$ 0.3125.

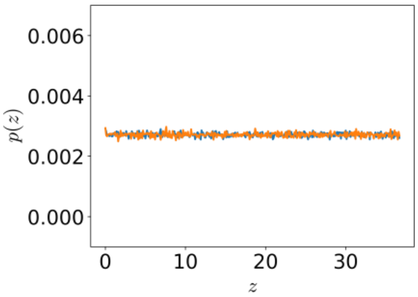

Figure S49: Directional density-distributions at reduced temperature $T^* =$ 2.8 for the system with reduced charge position $z_c^* = 0.3125$.

System with reduced charge position $z_c^* = 0.3125$ at $T^* = 2.4$ – nematic phase

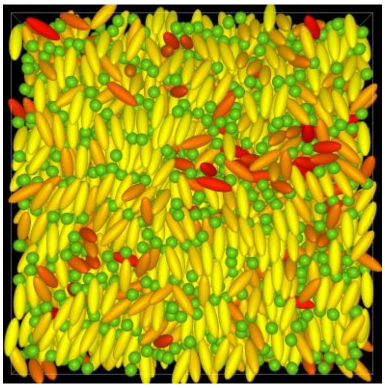

Figure S50: A picture of the 1000000th simulation snapshot at reduced temperature $T^* =$ 2.4 for the system with reduced charge position $z_c^* = 0.3125$. The system is in the nematic phase.

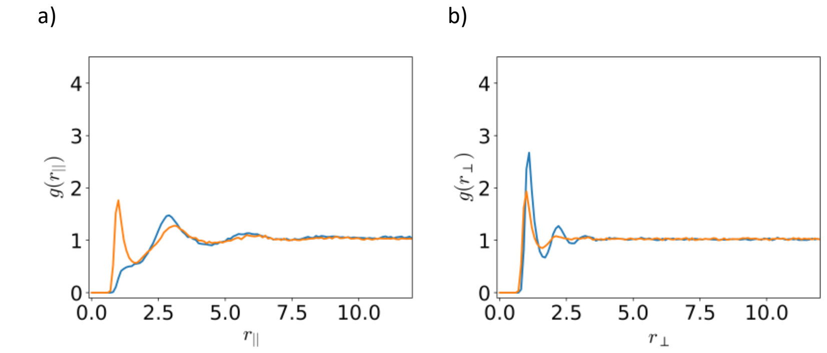

Figure S51: Directional pair correlation functions at reduced temperature $T^* =$ 2.4 for the system with reduced charge position $z_c^* =$ 0.3125. Calculated parallel (a) and orthogonal (b) to the director of the GB particles.

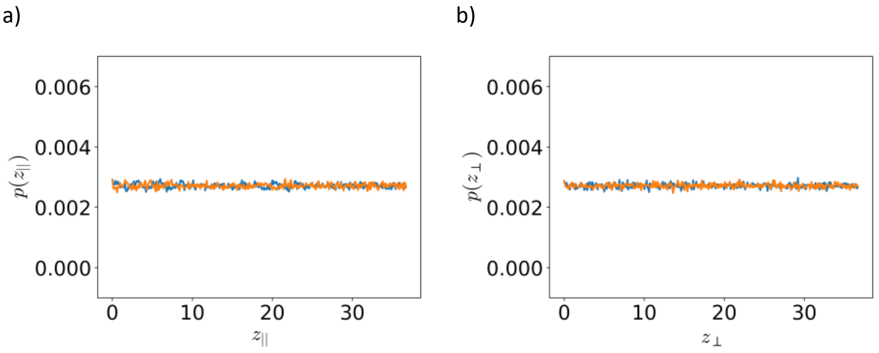

Figure S52: Directional density-distributions at reduced temperature $T^* =$ 2.4 for the system with reduced charge position $z_c^* =$ 0.3125. Calculated parallel (a) and orthogonal (b) to the director of the GB particles.

System with reduced charge position $\boldsymbol{z_c^*} = \mathbf{0.3125}$ at $\boldsymbol{T^*} = \mathbf{1.7}$ – smectic A phase

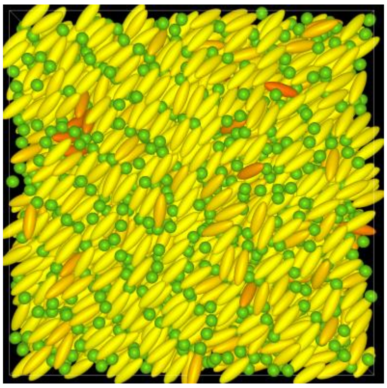

Figure S53: A picture of the 1000000th simulation snapshot at reduced temperature $T^* =$ 1.7 for the system with reduced charge position $z_c^* =$ 0.3125. The system is in the smectic A phase.

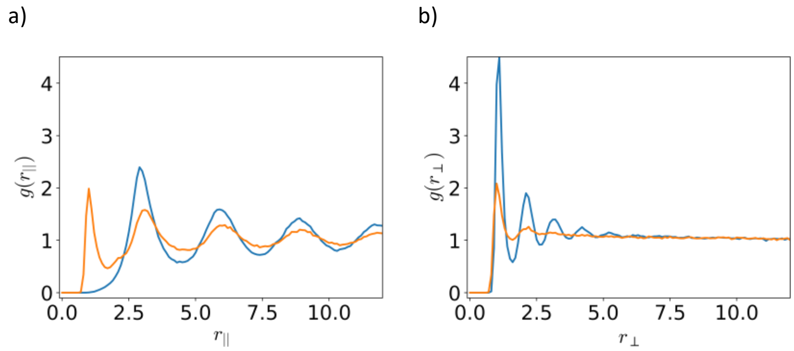

Figure S54: Directional pair correlation functions at reduced temperature $T^* = 1.7$ for the system with reduced charge position $z_c^* = 0.3125$. Calculated parallel (a) and orthogonal (b) to the director of the GB particles.

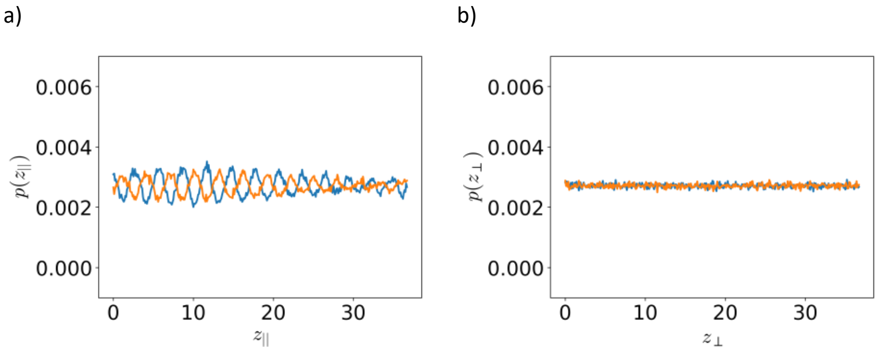

Figure S55: Directional density-distributions at reduced temperature $T^* = 1.7$ for the system with reduced charge position $z_c^* = 0.3125$. Calculated parallel (a) and orthogonal (b) to the director of the GB particles.

System with reduced charge position $\boldsymbol{z_c^* = 0.25}$ – overview

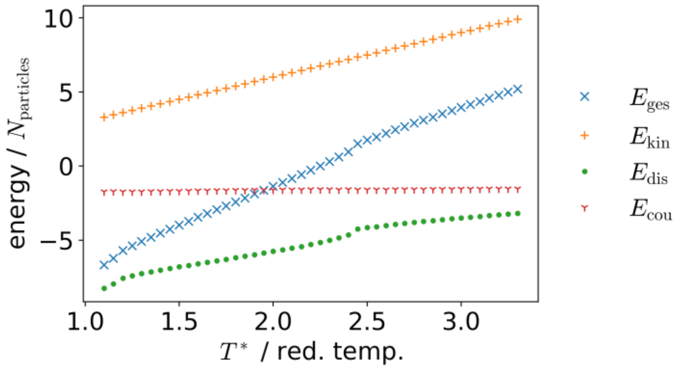

Figure S56: Energies over reduced temperature T^* for the system with reduced charge position $z_c^* = 0.25$.

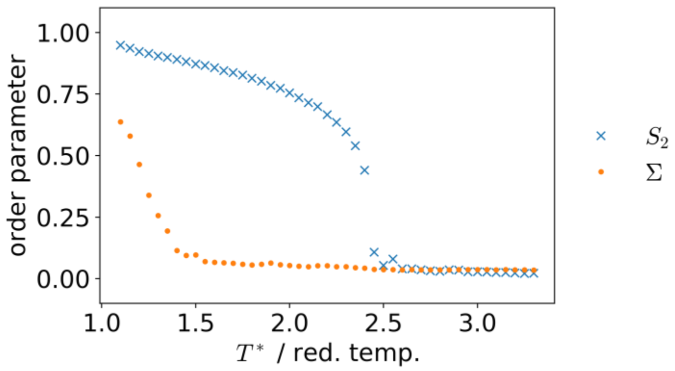

Figure S57: Orientational order parameter S_2 and translational order parameter Σ over reduced temperature T^* for the system with reduced charge position $z_c^* = 0.25$.

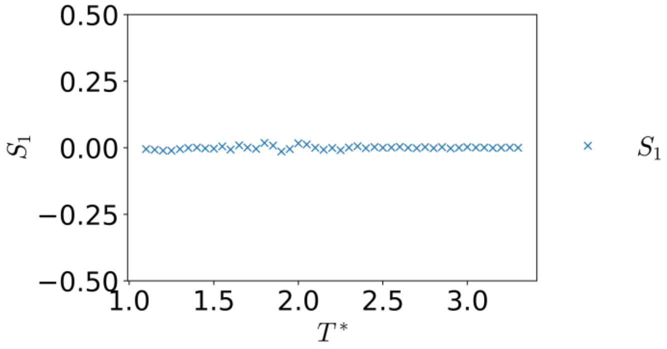

Figure S58: Polar order parameter S_1 over reduced temperature T^* for the system with reduced charge position $z_c^* = 0.25$.

System with reduced charge position $\boldsymbol{z_c^* = 0.25}$ at $\boldsymbol{T^* = 3.3}$ – isotropic phase

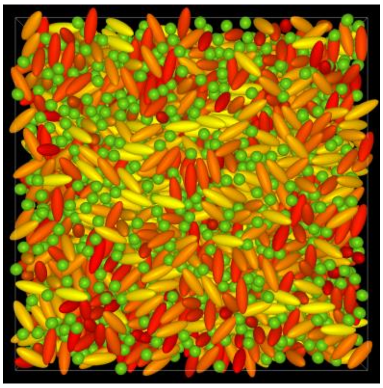

Figure S59: A picture of the 1000000th simulation snapshot at reduced temperature $T^* =$ 3.3 for the system with reduced charge position $z_c^* = 0.25$. The system is in the isotropic phase.

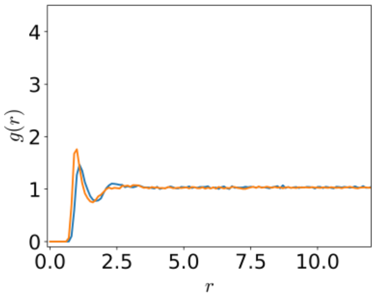

Figure S60: Directional pair correlation functions at reduced temperature $T^* =$ 3.3 for the system with reduced charge position $z_c^* = 0.25$.

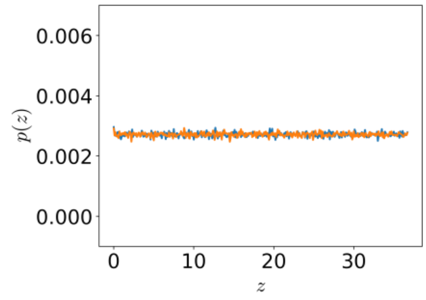

Figure S61: Directional density-distributions at reduced temperature $T^* =$ 3.3 for the system with reduced charge position $z_c^* = 0.25$.

System with reduced charge position $\boldsymbol{z_c^* = 0.25}$ at $\boldsymbol{T^* = 1.8}$ – nematic phase

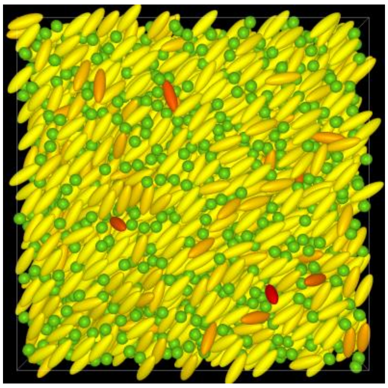

Figure S62: A picture of the 1000000th simulation snapshot at reduced temperature $T^* =$ 1.8 for the system with reduced charge position $z_c^* = 0.25$. The system is in the nematic phase.

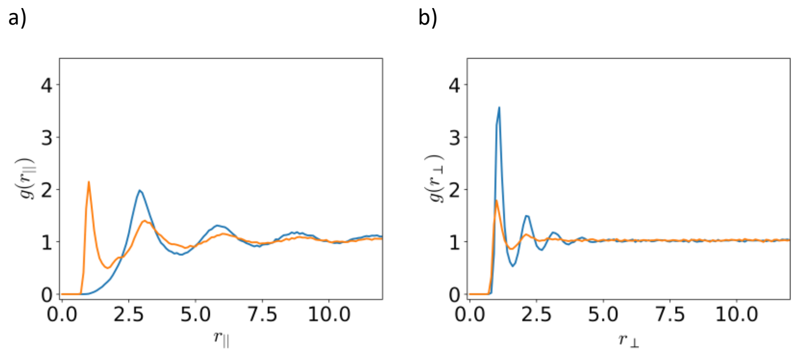

Figure S63: Directional pair correlation functions at reduced temperature $T^* =$ 1.8 for the system with reduced charge position $z_c^* = 0.25$. Calculated parallel (a) and orthogonal (b) to the director of the GB particles.

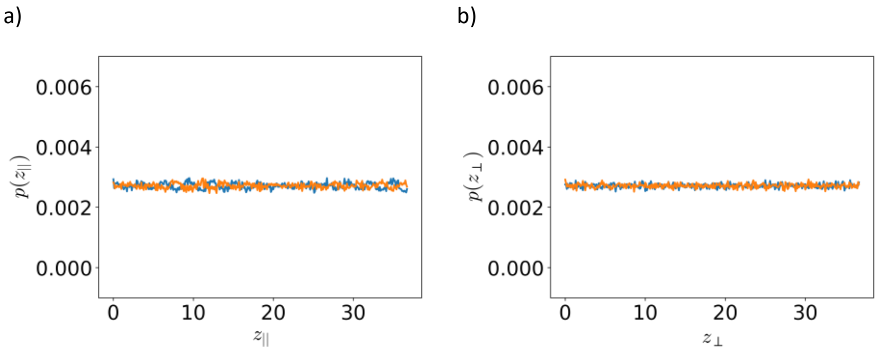

Figure S64: Directional density-distributions at reduced temperature $T^* =$ 1.8 for the system with reduced charge position $z_c^* = 0.25$. Calculated parallel (a) and orthogonal (b) to the director of the GB particles.

System with reduced charge position $\boldsymbol{z_c^* = 0.25}$ at $\boldsymbol{T^* = 1.35}$ – smectic phase

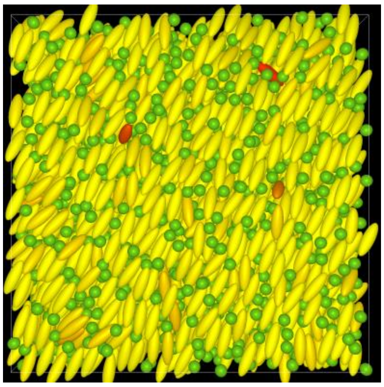

Figure S65: A picture of the 1000000th simulation snapshot at reduced temperature $T^* = 1.35$ for the system with reduced charge position $z_c^* = 0.25$. The system is in the nematic phase.

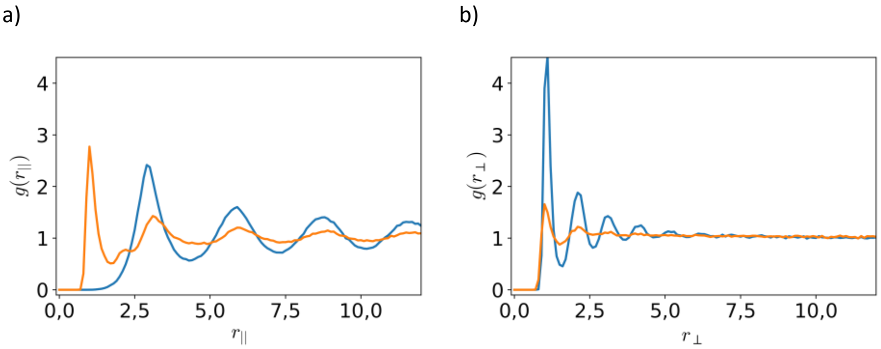

Figure S66: Directional pair correlation functions at reduced temperature $T^* = 1.35$ for the system with reduced charge position $z_c^* = 0.25$. Calculated parallel (a) and orthogonal (b) to the director of the GB particles.

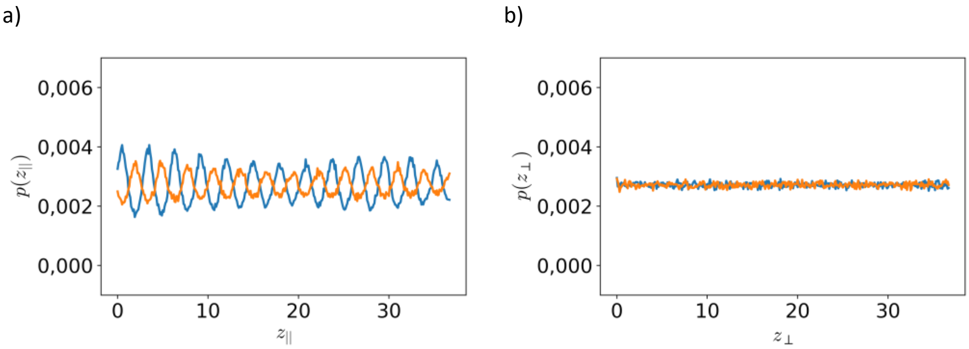

Figure S67: Directional density-distributions at reduced temperature $T^* = 1.35$ for the system with reduced charge position $z_c^* = 0.25$. Calculated parallel (a) and orthogonal (b) to the director of the GB particles.

System with reduced charge position $\boldsymbol{z_c^*} = \mathbf{0.25}$ at $\boldsymbol{T^*} = \mathbf{1.15}$ – crystalline phase

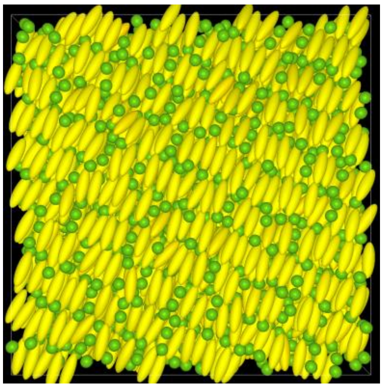

Figure S68: A picture of the 1000000th simulation snapshot at reduced temperature $T^* = 1.15$ for the system with reduced charge position $z_c^* = 0.25$. The system is in the nematic phase.

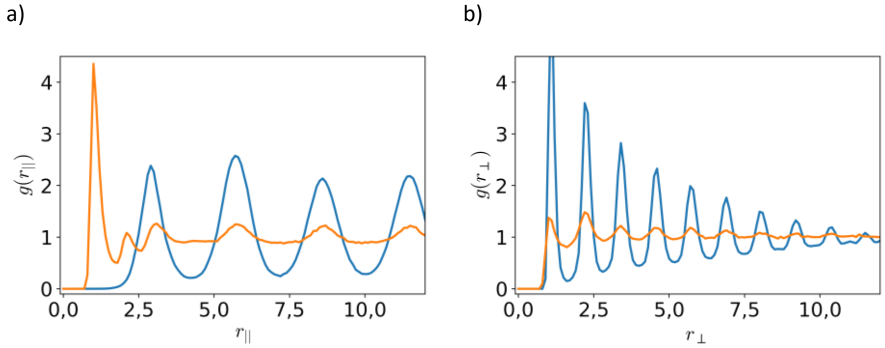

Figure S69: Directional pair correlation functions at reduced temperature $T^* = 1.15$ for the system with reduced charge position $z_c^* = 0.25$. Calculated parallel (a) and orthogonal (b) to the director of the GB particles.

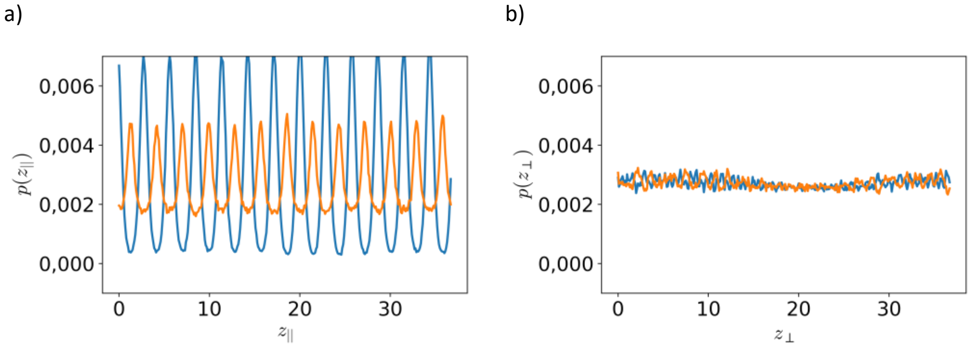

Figure S70: Directional density-distributions at reduced temperature $T^* = 1.15$ for the system with reduced charge position $z_c^* = 0.25$. Calculated parallel (a) and orthogonal (b) to the director of the GB particles.

System with reduced charge position $z_c^* = 0.125$ – overview

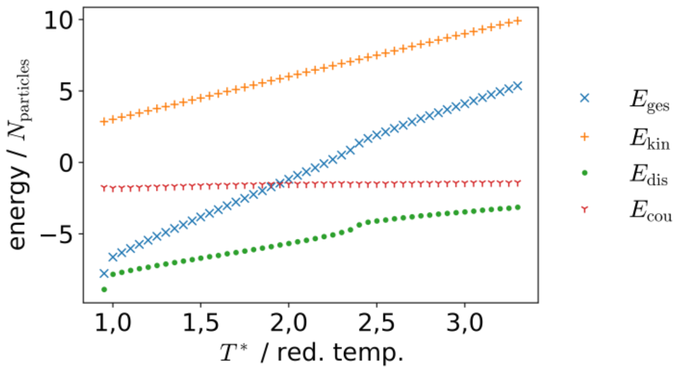

Figure S71: Energies over reduced temperature T^* for the system with reduced charge position $z_c^* = 0.125$.

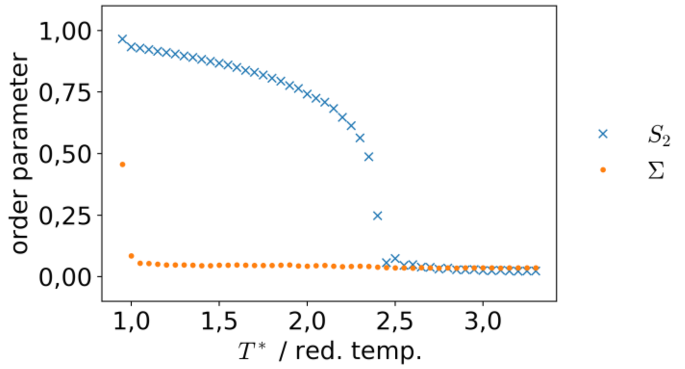

Figure S72: Orientational order parameter S_2 and translational order parameter Σ over reduced temperature T^* for the system with reduced charge position $z_c^* = 0.125$.

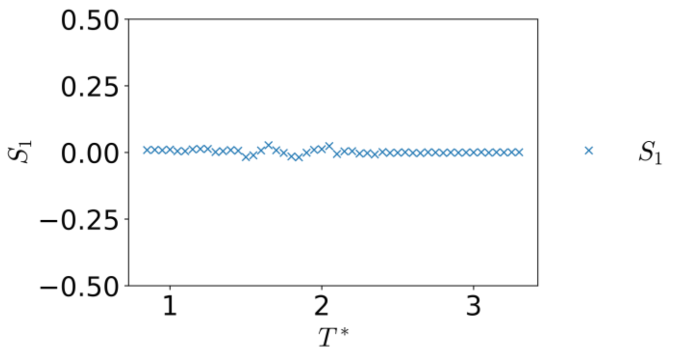

Figure S73: Polar order parameter S_1 over reduced temperature T^* for the system with reduced charge position $z_c^* = 0.125$.

System with reduced charge position $z_c^* = 0.125$ at $T^* = 3.3$ – isotropic phase

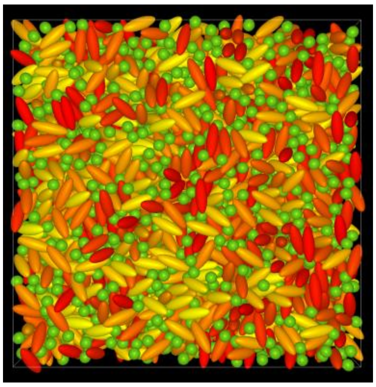

Figure S74: A picture of the 1000000th simulation snapshot at reduced temperature $T^* =$ 3.3 for the system with reduced charge position $z_c^* = 0.125$. The system is in the isotropic phase.

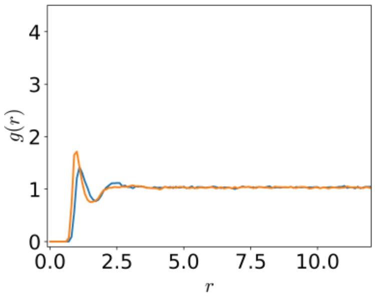

Figure S75: Directional pair correlation functions at reduced temperature $T^* =$ 3.3 for the system with reduced charge position $z_c^* = 0.125$.

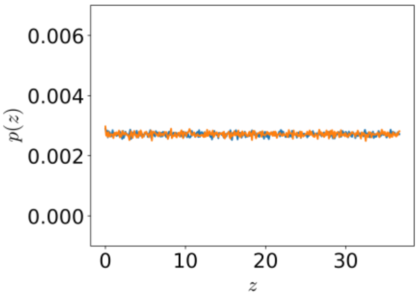

Figure S76: Directional density-distributions at reduced temperature $T^* =$ 3.3 for the system with reduced charge position $z_c^* = 0.125$.

System with reduced charge position $z_c^* = 0.125$ at $T^* = 2.2$ – nematic phase

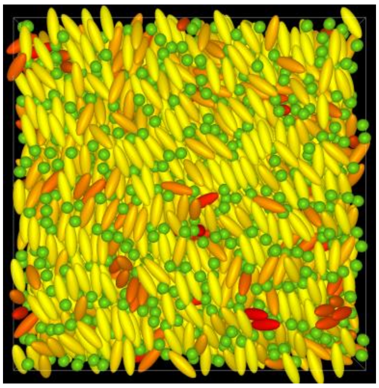

Figure S77: A picture of the 1000000th simulation snapshot at reduced temperature $T^* =$ 2.2 for the system with reduced charge position $z_c^* = 0.125$. The system is in the nematic phase.

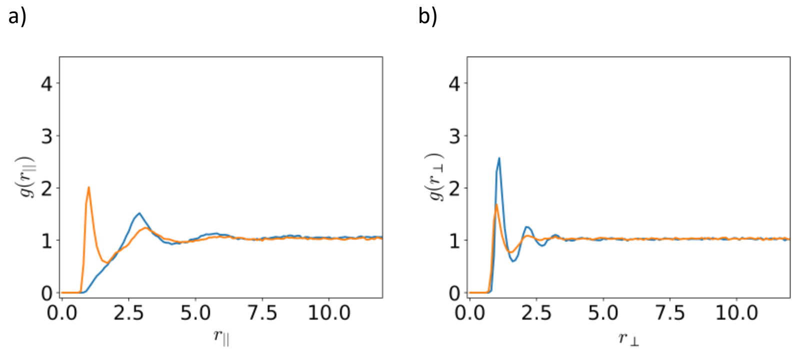

Figure S78: Directional pair correlation functions at reduced temperature $T^* =$ 2.2 for the system with reduced charge position $z_c^* = 0.125$. Calculated parallel (a) and orthogonal (b) to the director of the GB particles.

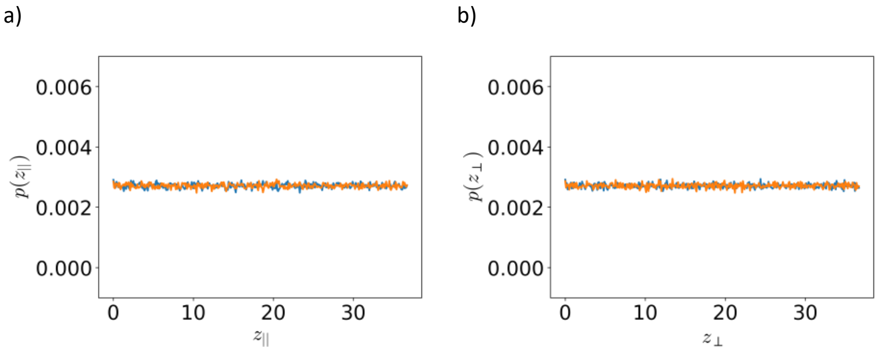

Figure S79: Directional density-distributions at reduced temperature $T^* =$ 2.2 for the system with reduced charge position $z_c^* = 0.125$. Calculated parallel (a) and orthogonal (b) to the director of the GB particles.

System with reduced charge position $\boldsymbol{z_c^* = 0.125}$ at $\boldsymbol{T^* = 0.95}$ – nematic phase

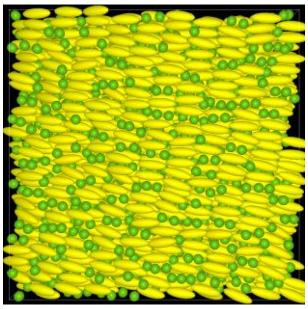

Figure S80: A picture of the 1000000th simulation snapshot at reduced temperature $T^* =$ 0.95 for the system with reduced charge position $z_c^* = 0.125$. The system is in the nematic phase.

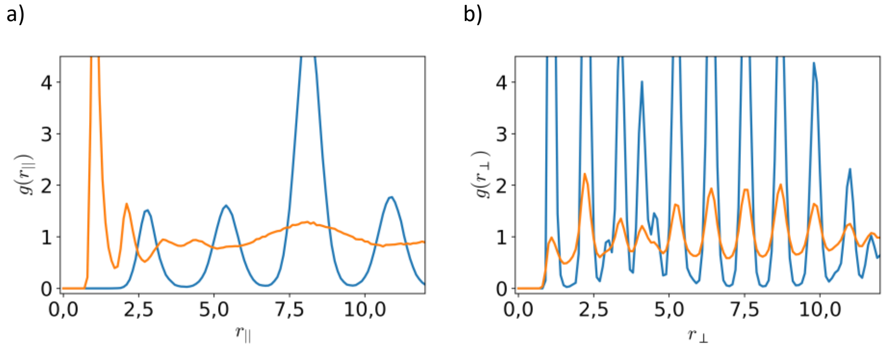

Figure S81: Directional pair correlation functions at reduced temperature $T^* =$ 0.95 for the system with reduced charge position $z_c^* = 0.125$. Calculated parallel (a) and orthogonal (b) to the director of the GB particles.

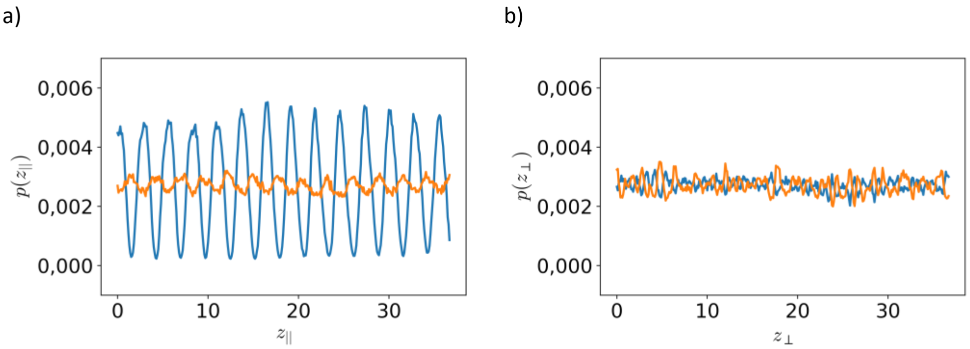

Figure S82: Directional density-distributions at reduced temperature $T^* = 0.95$ for the system with reduced charge position $z_c^* = 0.125$. Calculated parallel (a) and orthogonal (b) to the director of the GB particles.

System with reduced charge position $z_c^* = 0.0$ – overview

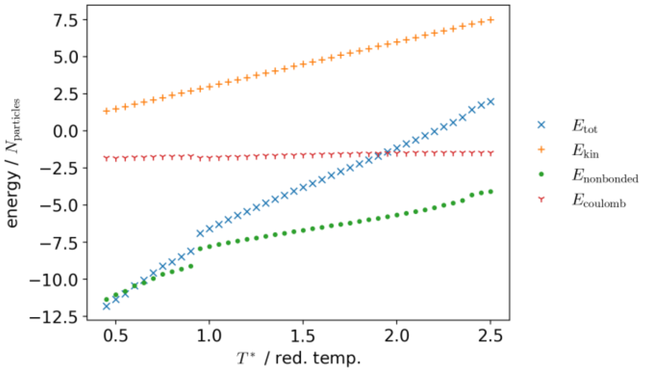

Figure S83: Energies over reduced temperature T^* for the system with reduced charge position $z_c^* = 0.0$.

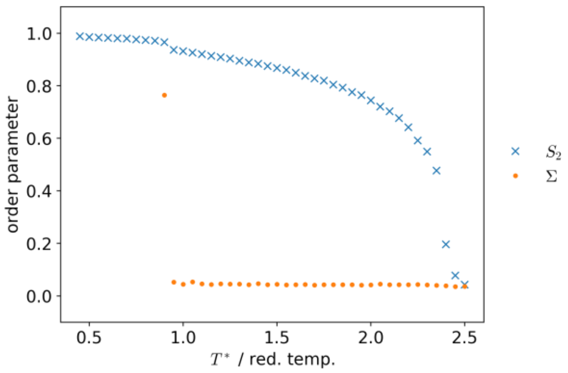

Figure S84: Orientational order parameter S_2 and translational order parameter Σ over reduced temperature T^* for the system with reduced charge position $z_c^* = 0.0$. The system transitions into a crystalline phase starting at $T^* = 0.9$ and at this point the system has free volume. Due to the free volume the calculation of the translational order parameter is unreliable for temperatures lower than $T^* = 0.9$.

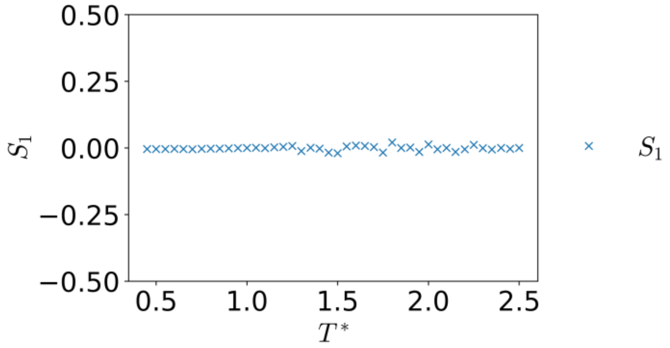

Figure S85: Polar order parameter S_1 over reduced temperature T^* for the system with reduced charge position $z_c^* = 0.0$.

System with reduced charge position $\boldsymbol{z_c^*} = \mathbf{0.0}$ at $\boldsymbol{T^*} = \mathbf{2.5}$ – isotropic phase

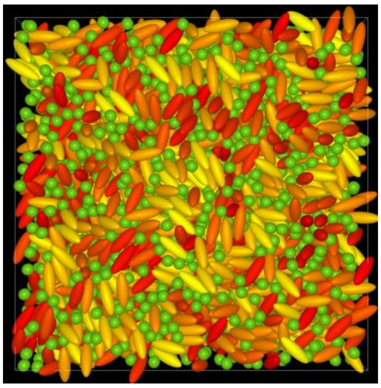

Figure S86: A picture of the 1000000th simulation snapshot at reduced temperature $T^* = 2.5$ for the system with reduced charge position $z_c^* = 0.0$. The system is in the isotropic phase.

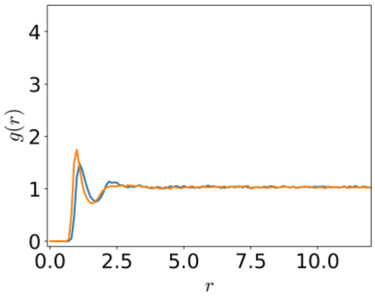

Figure S87: Directional pair correlation functions at reduced temperature $T^* = 2.5$ for the system with reduced charge position $z_c^* = 0.0$.

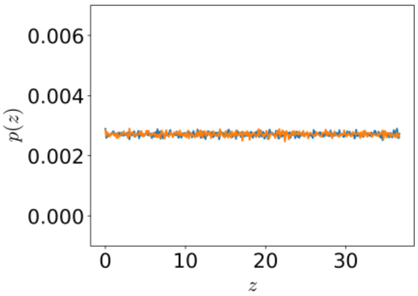

Figure S88: Directional density-distributions at reduced temperature $T^* = 2.5$ for the system with reduced charge position $z_c^* = 0.0$.

System with reduced charge position $\boldsymbol{z_c^*} = \mathbf{0.0}$ at $\boldsymbol{T^*} = \mathbf{2.0}$ – nematic phase

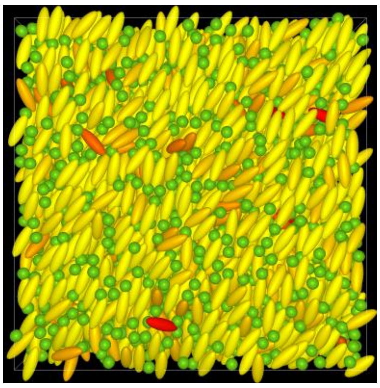

Figure S89: A picture of the 1000000th simulation snapshot at reduced temperature $T^* = 2.0$ for the system with reduced charge position $z_c^* = 0.0$. The system is in the nematic phase.

a) b)

#

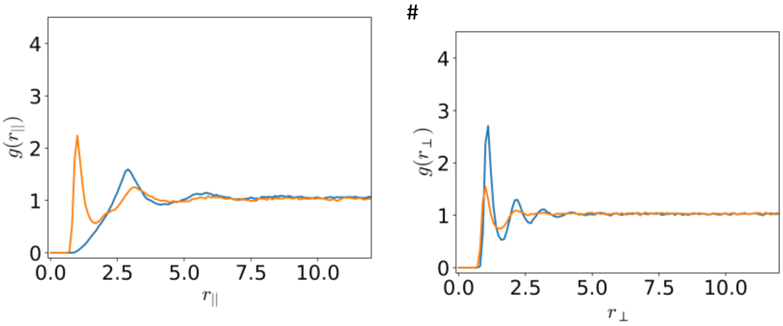

Figure S90: Directional pair correlation functions at reduced temperature $T^* =$ 2.0 for the system with reduced charge position $z_c^* = 0.0$. Calculated parallel (a) and orthogonal (b) to the director of the GB particles.

a) b)

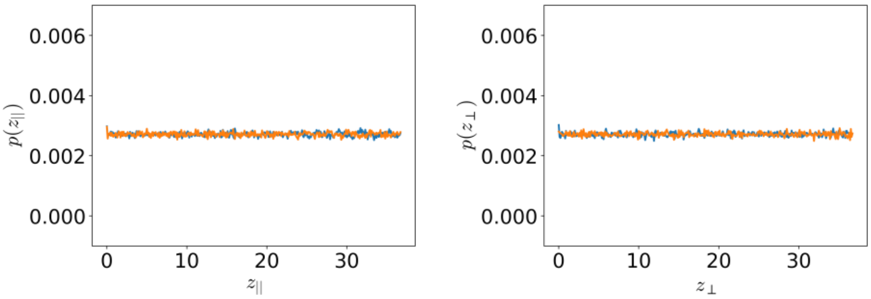

Figure S91: Directional density-distributions at reduced temperature $T^* =$ 2.0 for the system with reduced charge position $z_c^* = 0.0$. Calculated parallel (a) and orthogonal (b) to the director of the GB particles.

System with reduced charge position $\boldsymbol{z_c^* = 0.0}$ at $\boldsymbol{T^* = 0.9}$ – crystalline phase

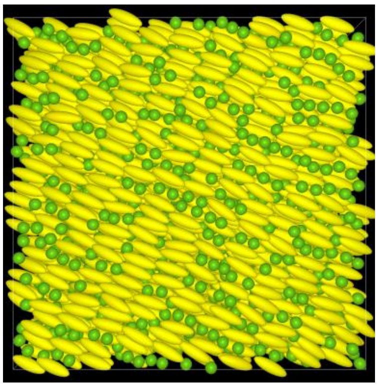

Figure S92: A picture of the 1000000th simulation snapshot at reduced temperature $T^* =$ 0.9 for the system with reduced charge position $z_c^* = 0.0$. The system is in a crystalline phase.

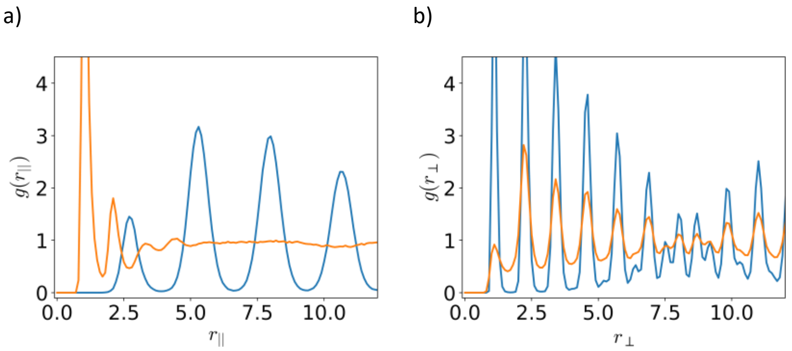

Figure S93: Directional pair correlation functions at reduced temperature $T^* = 0.9$ for the system with reduced charge position $z_c^* = 0.0$. Calculated parallel (a) and orthogonal (b) to the director of the GB particles.

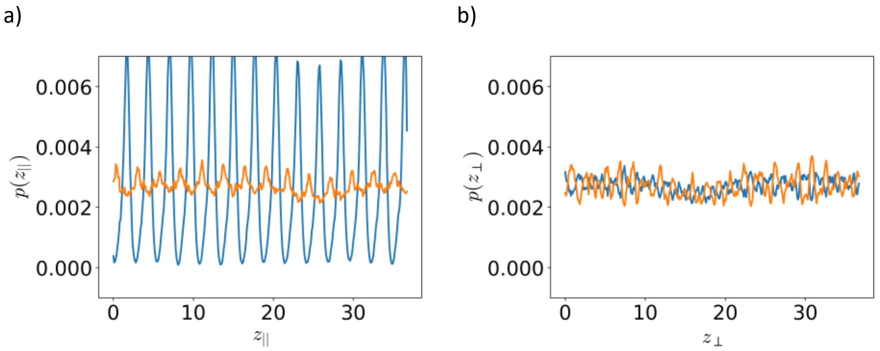

Figure S94: Directional density-distributions at reduced temperature $T^* = 0.9$ for the system with reduced charge position $z_c^* = 0.0$. Calculated parallel (a) and orthogonal (b) to the director of the GB particles.

References

[S1] J. G. Gay, B. J. Berne, *J. Chem. Phys.* **1981**, *74*, 3316.

[S2] F. Weik, R. Weeber, K. Szuttor, K. Breitsprecher, J. de Graaf, M. Kuron, J. Landsgesell, H. Menke, D. Sean, C. Holm, *Eur. Phys. J. Spec. Top.* **2019**, *227*, 1789.

[S3] a) R. W. Hockney, J. W. Eastwood, *Computer Simulation using particles*, Hilger, Bristol, **1988**; b) J. Kolafa, J. W. Perram, *Mol. Simul.* **1992**, *9*, 351.

[S4] R. Berardi, A. P. J. Emerson, C. Zannoni, *J. Chem. Soc. Faraday Trans.* **1993**, *89*, 4069.

[S5] G. Saielli, T. Margola, K. Satoh, *Soft matter* **2017**, *13*, 5204.

[S6] H. A. Lorentz, *Ann. Phys.* **1881**, *248*, 127.

[S7] D. Berthelot, *C. R. Acad. Sci.*, *126*, 1703.

[S8] M. P. Allen, D. J. Tildesley, *Computer simulation of liquids*, Clarendon Press, Oxford, **1990**.
